# Supplementary material for: NTCP ubiquitination enables HBV infection
Source: JHEP Rep. 2025 Jul 31;7(11):101534. doi: 10.1016/j.jhepr.2025.101534 (PMC12538160; doi:10.1016/j.jhepr.2025.101534)
Supplement: Multimedia component 1 [file mmc1.pdf]

NTCP ubiquitination enables HBV infection

Monique D. Appelman, Thuc-Anh Nguyen, Andreas Oswald, Aaron Lucko, Coen C. Paulusma, Ulrike Protzer, Stan F.J. van de Graaf

Table of contents

Fig. S1……………………………………………………………………………………2

Fig. S2……………………………………………………………………………………4

Fig. S3……………………………………………………………………………………5

Fig. S4……………………………………………………………………………………7

Fig. S5……………………………………………………………………………………8

Fig. S6……………………………………………………………………………………9

Fig. S7……………………………………………………………………………………10

Table S1…………………………………………………………………………………11

Table S2…………………………………………………………………………………11

A

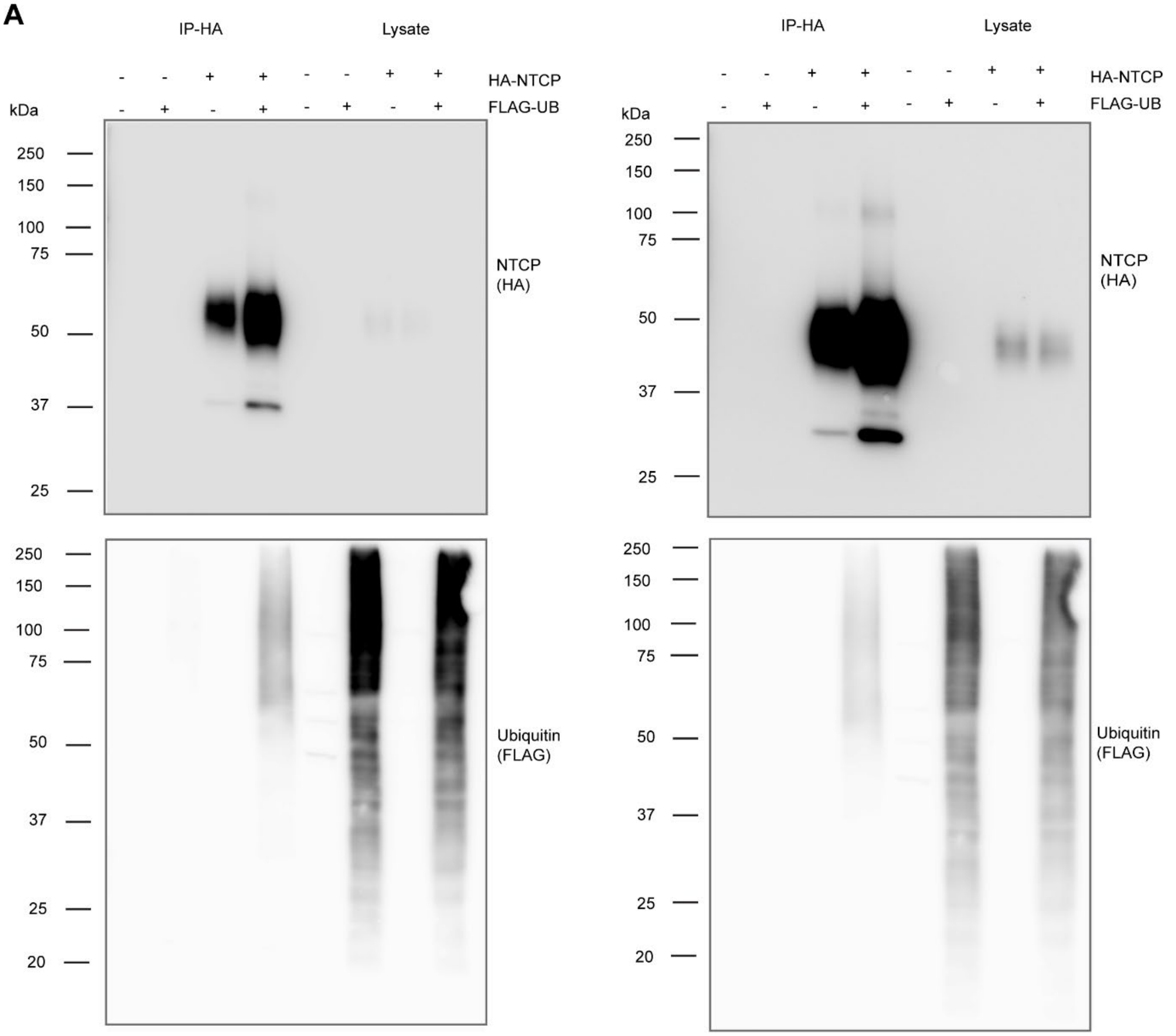
IP-HA
Lysate
HA-NTCP
FLAG-UB
kDa
250
150
100
75
50
37
25
20
NTCP (HA)
Ubiquitin (FLAG)

B

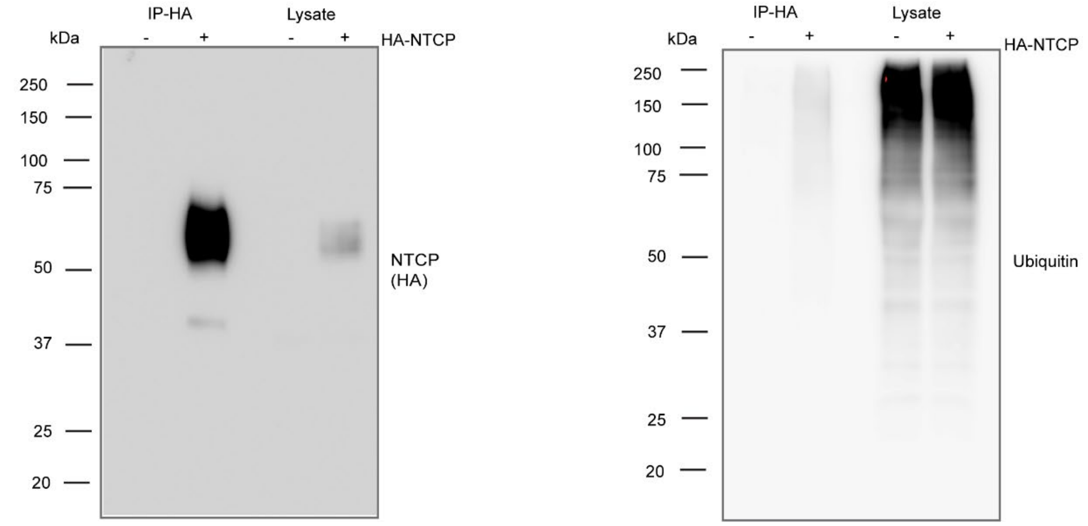
IP-HA
Lysate
HA-NTCP
kDa
250
150
100
75
50
37
25
20
NTCP (HA)
Ubiquitin

Fig. S1. Complete uncropped western blots of Fig. 1. (A) Complete western blot of Fig. 1A showing FLAG-Ubiquitin co-precipitation with HA-NTCP in U2OS cells stably expressing HA-tagged NTCP and transient overexpressing FLAG-tagged ubiquitin in two different light intensities. (B) Complete western blot of Fig. 1B showing endogenous ubiquitin co-precipitation with HA-NTCP in U2OS cells.

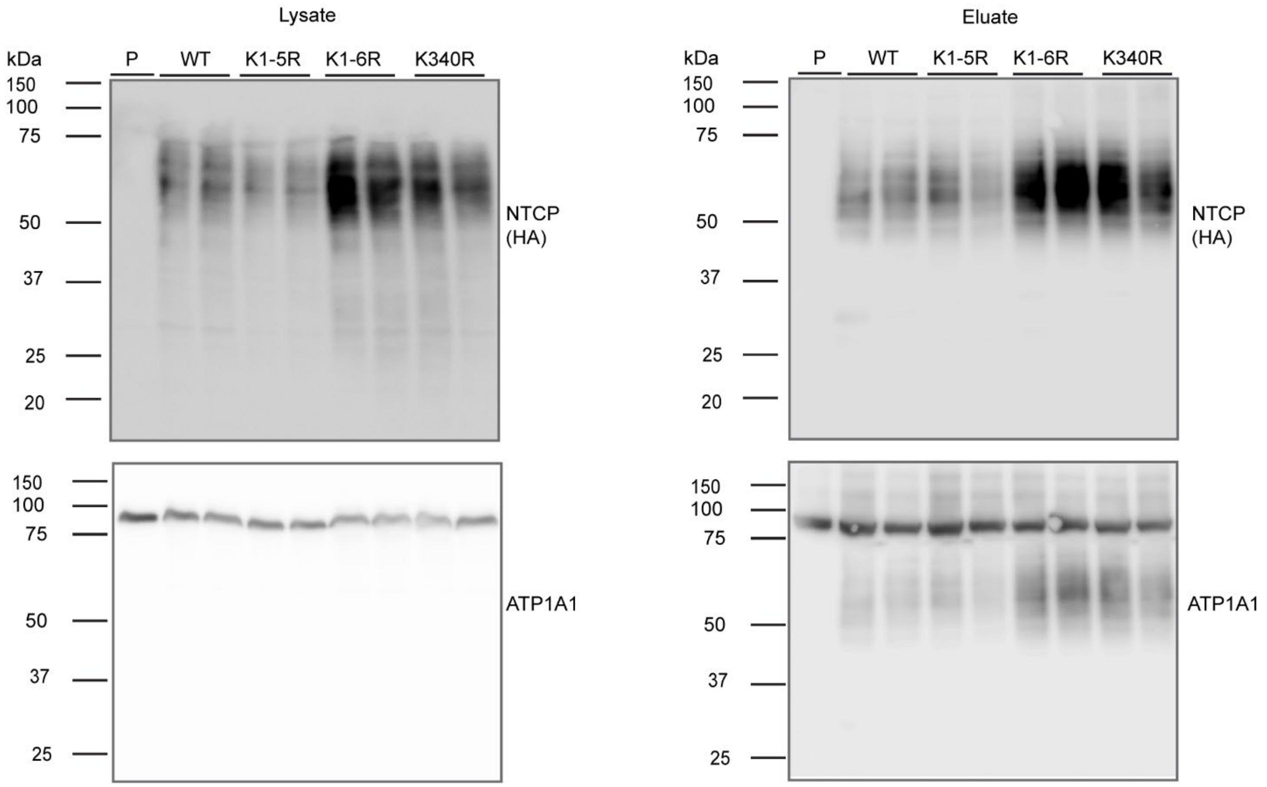

Fig. S2. Complete uncropped western blots of Fig.2. Complete western blot of Fig 2D showing immunoblot from a surface biotinylation experiment of HepG2 cells stably expressing $NTCP^{WT}$, $NTCP^{K1\text{-}5R}$, $NTCP^{K1\text{-}6R}$ and $NTCP^{K340R}$ showing NTCP protein levels in total lysate (left) and at the plasma membrane (right). ATP1A1 was included as a transfer control. ‘P’ denotes parental cells without HA-NTCP overexpression.

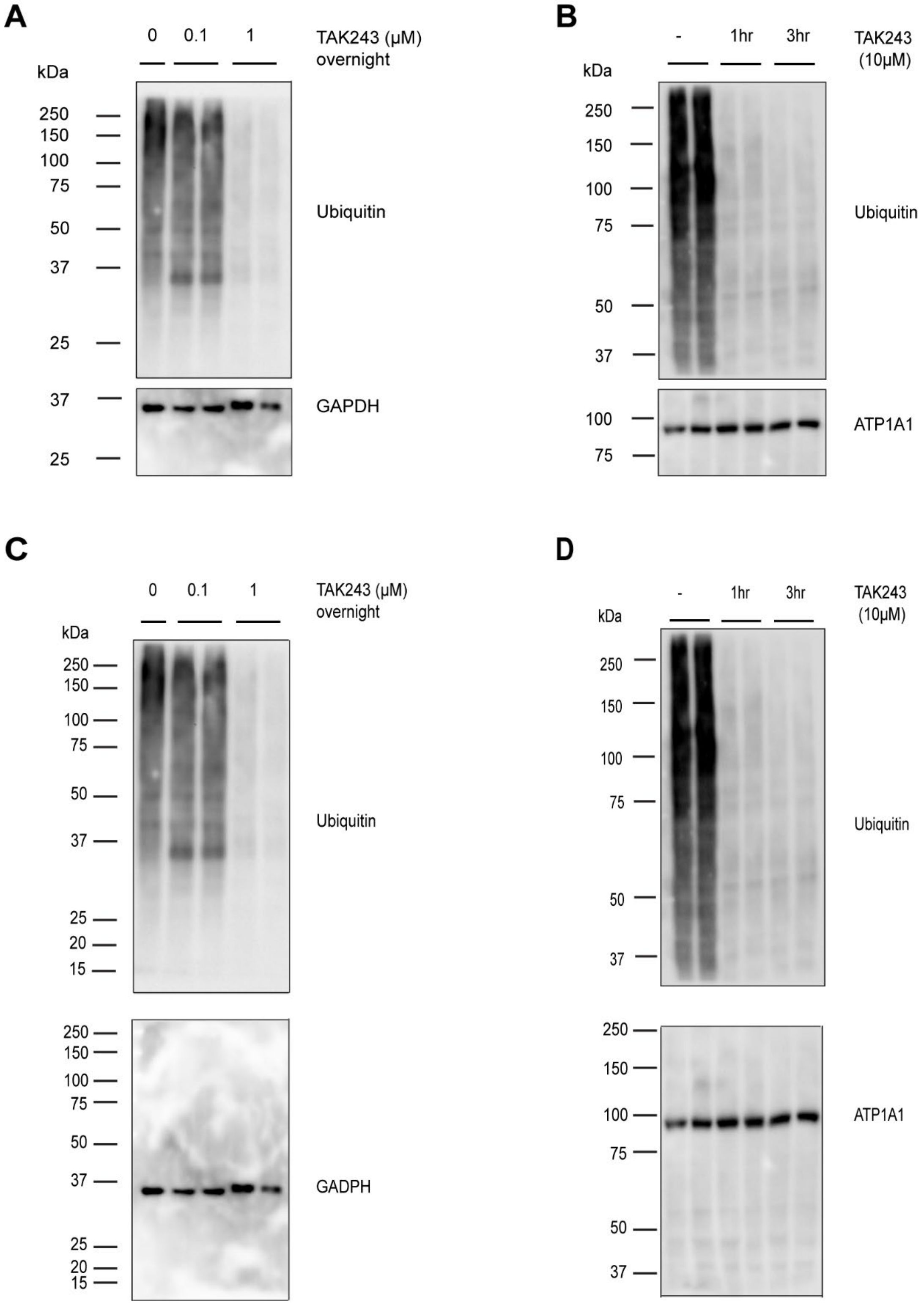

Fig. S3. Ubiquitination inhibition with TAK-243. Cropped (A, B) and uncropped (C, D) western blots of HepG2 cells overexpressing NTCP^{WT} treated withTAK-243. Overnight treatment of TAK-243 reduces ubiquitination at 0.1 μM but most efficiently at 1 μM (A, C).

At a high concentration of 10 μM, TAK-243 reduces ubiquitination markedly after 1-3 hours of treatment (B, D).

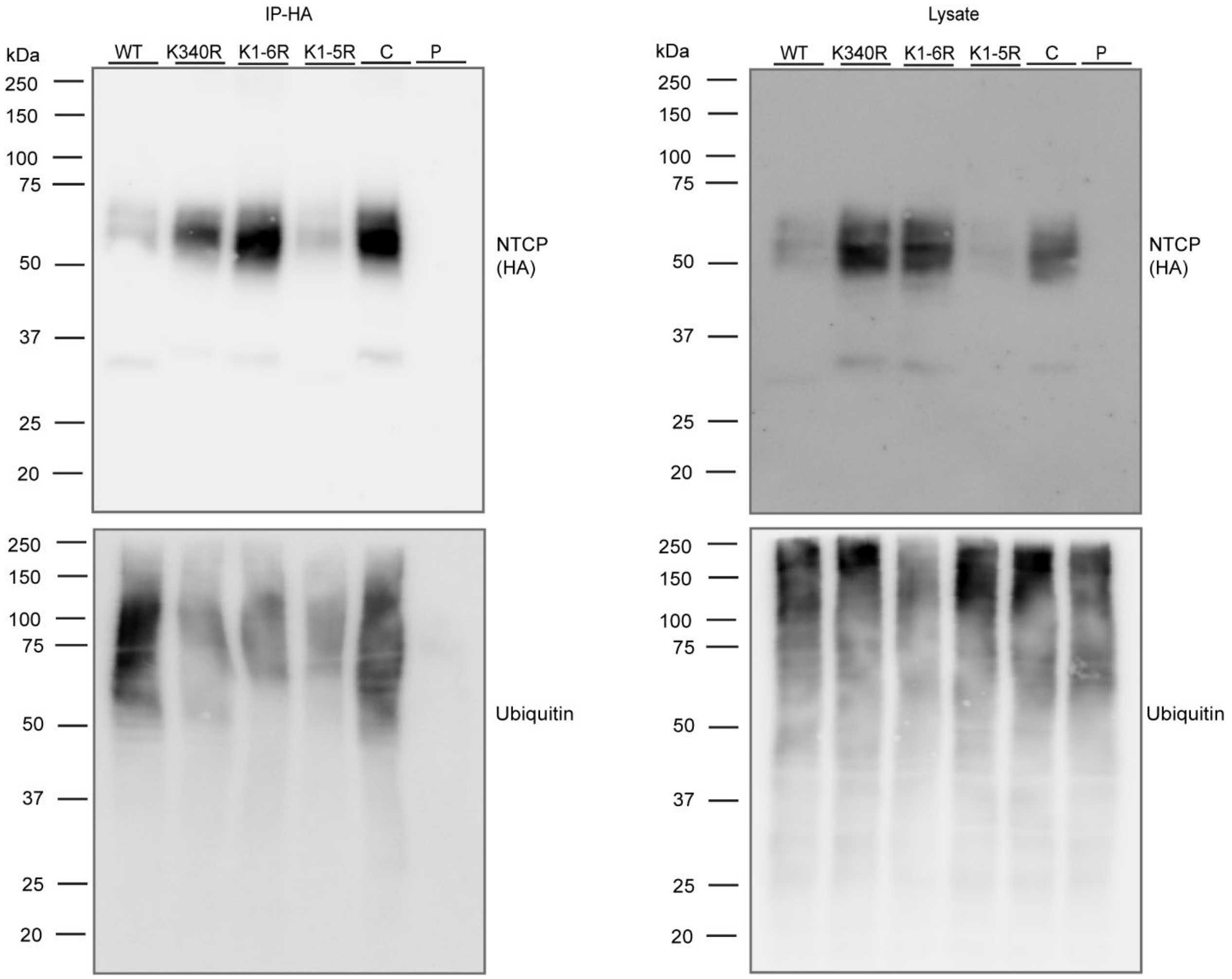

Fig. S4. Complete uncropped western blot of Fig 2G. Complete western blot of Fig. 2G showing immunoprecipitation of HA-NTCP in U2OS cells stably expressing HA-$NTCP^{WT}$ or HA-$NTCP^{K340R}$, $NTCP^{K1\text{-}5R}$, $NTCP^{K1\text{-}6R}$, HA-NTCP (C) or parental U2OS (P), which shows that co-precipitation of ubiquitin in HA-$NTCP^{K340R}$ cells was strongly reduced compared to HA $NTCP^{WT}$ cells. The HA-NTCP (C) serves as a positive control in this experiment. Western blot is cropped and flipped horizontally to generate Fig. 2G.

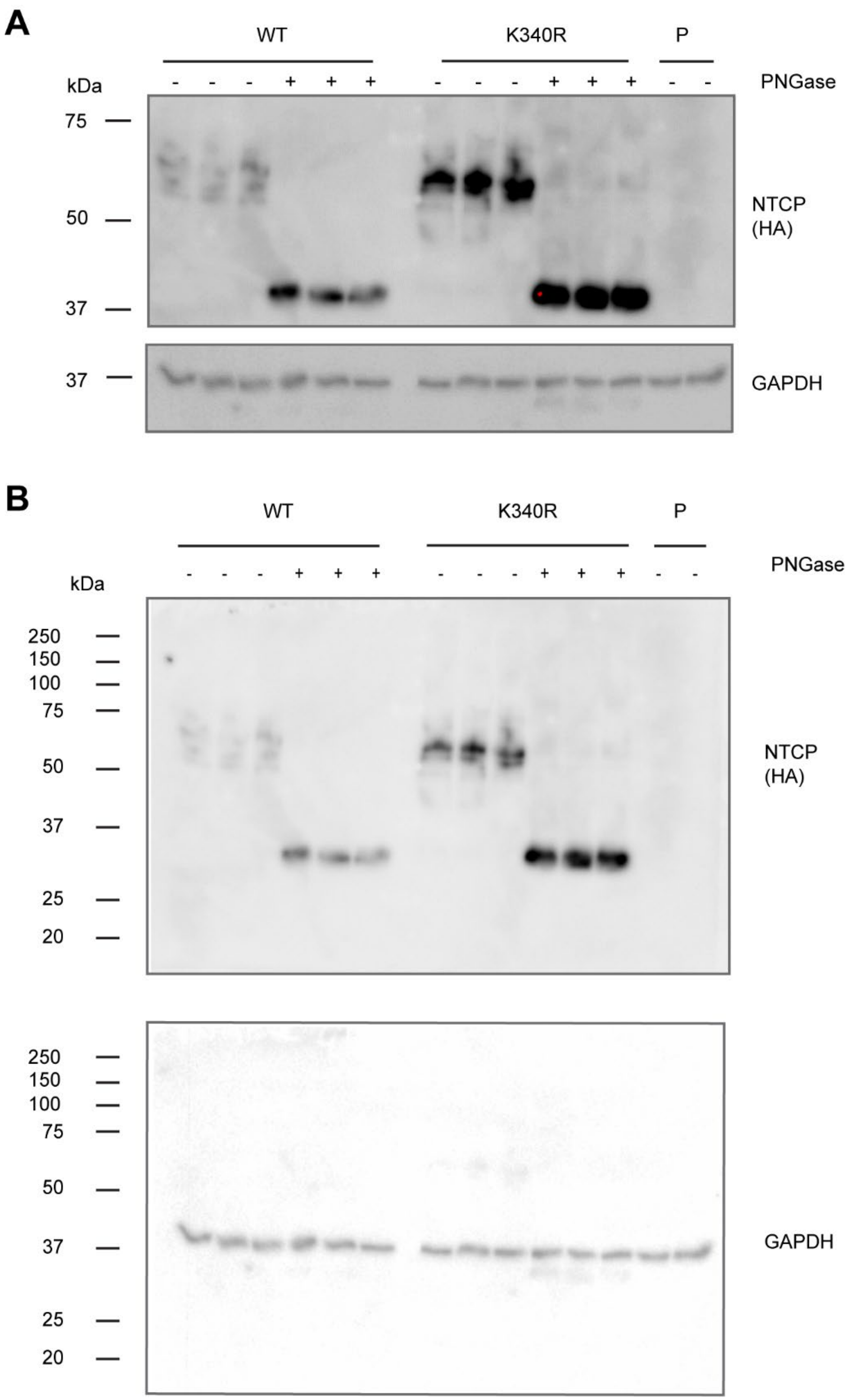

Fig. S5. The K340R mutation does not affect NTCP glycosylation. Cropped (A) and complete uncropped (B) western blots of lysates of HepG2 cells overexpressing $NTCP^{WT}$ or $NTCP^{K340R}$ or parental treated with PNGaseF for 2h at 37°C (500 units) prior to immunoblotting for NTCP.

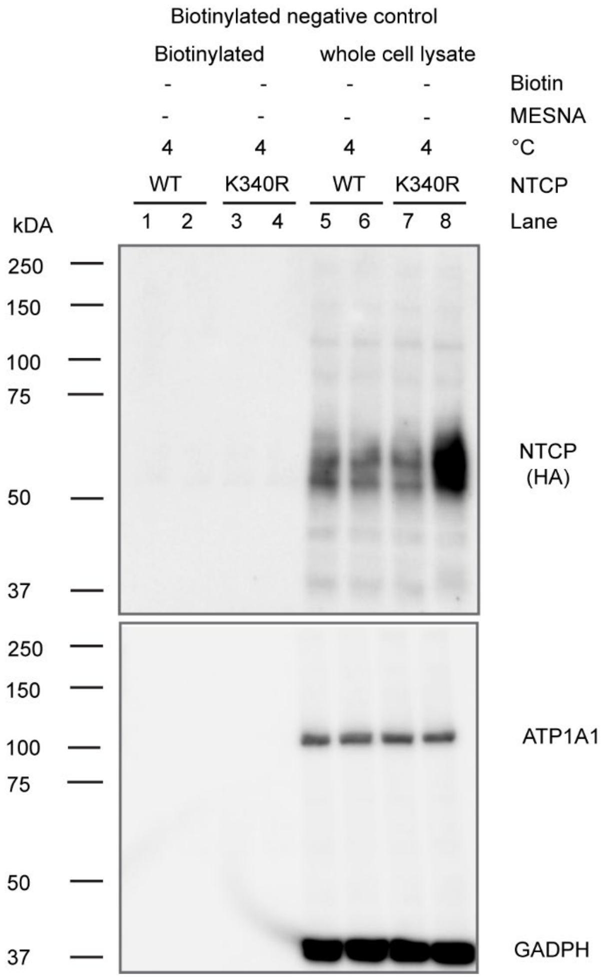

Fig. S6. Negative controls for biotinylation. Non-biotinylated fractions of HepG2 cells stably expressing $NTCP^{WT}$ or $NTCP^{K340R}$ were quantified**.** The omission of biotin prevented the precipitation of NTCP while in whole-cell lysate, NTCP was detected.

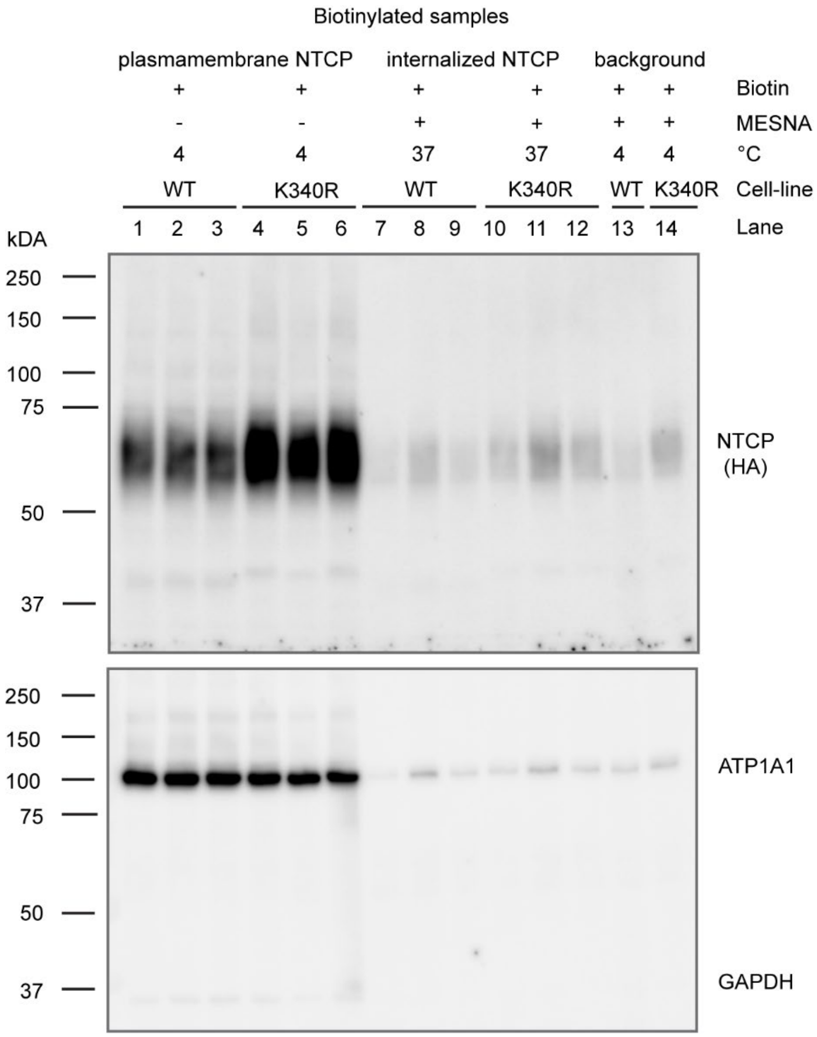

Fig. S7. Complete uncropped western blot of Fig. 3B. Endocytosis of $NTCP^{WT}$ and $NTCP^{K340R}$ in HepG2 cells was examined using biotin pulse-chase labeling of plasma membrane proteins. Total plasma membrane NTCP was determined from biotinylated cells kept at 4°C. To quantify internalized NTCP, cells were incubated at 37°C for 1.5 hour to allow endocytosis of biotinylated NTCP followed by MESNA treatment to remove biotin remaining on the plasma membrane. Additionally, biotinylated cells kept at 4°C were treated with MESNA to examine MESNA efficiency and quantify background fractions.

Supplementary Tables

Table S1. Primer pairs used for generating HA-hNTCP lysine mutants

Construct	Primers
HA-hNTCP^{K1-5R}	Fw: TGGTGCTATGAGAGATTCAGGACTCCCAGGGATAGAACAAGAATGATCTACACA<br>Rv: TGTGTATAGCATTCTTGTTCTATCCCTGGGAGTCCTGAATCTCTCATAGCACCA
HA-hNTCP^{K340R}	Fw: GCTCTGGGAAATGGCACCTACAGAGGGAGGACTGCTCC<br>Rv: GGAGCAGTCCTCCCCTCTGTAGGTGCCATTTCCCAG
Seq-hNTCP 1	CATGAAGGGGGACATGAACCTC
Seq-hNTCP 2	TGATGCCTTTTATTGGCTTT

Table S2. Primers used for qRT-PCR

Gene	Primers
NTCP	Fw: GGACATGAACCTCAGCATTGTG<br>Rv:GCCGTTTGGATTTGAGGACG
36B4	Fw:TCATCAACGGTACAAACGA<br>Rv: GAACGACTTTTCCAGTTCCG
HBV cccDNA	Fw: GACTCTCTCGTCCCCTTCTC<br>Rv: ATGGTGAGGTGAACAATGCT
HBV DNA, rcDNA	Fw: GTTGCCCGTTTGTCCTCTAATTC<br>Rv: GGAGGGATACATAGAGGTTCCTTGA
PrP	Fw: TGCTGGGAAGTGCCATGAG<br>Rv: CGGTGCATGTTTTCACGATAGTA
