# Supplementary material for: NTCP ubiquitination enables HBV infection
Source: JHEP Rep. 2025 Jul 31;7(11):101534. doi: 10.1016/j.jhepr.2025.101534 (PMC12538160; doi:10.1016/j.jhepr.2025.101534)
Supplement: Multimedia component 2 [file mmc2.docx]

**JHEP Reports**

**CTAT methods**

Tables for a “Complete, Transparent, Accurate and Timely account” (CTAT) are now mandatory for all revised submissions. The aim is to enhance the reproducibility of methods.

- Only include the parts relevant to your study
- Refer to the CTAT in the main text as ‘Supplementary CTAT Table’
- Do not add subheadings
- Add as many rows as needed to include all information
- Only include one item per row

**If the CTAT form is not relevant to your study, please outline the reasons why:**

|  |
| --- |

- 1. **Antibodies**

| **Name** | **Citation** | **Supplier** | **Cat no.** | **Clone no.** |
| --- | --- | --- | --- | --- |
| ATP1A1 | Koenderink JB et al. J Biol Chem. 2003; 278: 51213-22 | Gift from JB Koenderink |  |  |
| GAPDH | - | Cell signalling, CST 2118S |  |  |
| anti-HA-HRP | - | Sigma, H6533 |  |  |
| Rabbit anti-FLAG | - | Sigma, F7425 |  |  |
| Mouse anti-FLAG | - | Sigma, F1804 |  |  |
| anti-mouse IgG HRP-conjugated | - | DAKO, P0447 |  |  |
| anti-rabbit IgG HRP-conjugated | - | Thermo scientific, 31460 |  |  |
| Ubiquitin | - | ENZO, BML-PW0150 |  |  |

- 1. **Cell lines**

| **Name** | **Citation** | **Supplier** | **Cat no.** | **Passage no.** | **Authentication test method** |
| --- | --- | --- | --- | --- | --- |
| HepG2 |  | ATCC, VA, USA | NA | Below 20 |  |
| U2OS |  | ATCC, VA, USA | NA | Below 20 |  |
| HEK293 |  | ATCC, VA, USA | NA | Below 20 |  |
| HepaRG | Gripon et al. Proc Natl  Acad Sci U S A. 2002 Nov 26;99 (24):15655-60. doi:10.1073/pnas.232137699.  Epub 2002 Nov 13. | Isolated in the lab of C. Trepo/F. Zoulim | NA | Below 20 |  |

- 1. **Organisms**

| **Name** | **Citation** | **Supplier** | **Strain** | **Sex** | **Age** | **Overall n number** |
| --- | --- | --- | --- | --- | --- | --- |
|  |  |  |  |  |  |  |

- 1. **Sequence based reagents**

| **Name** | **Sequence** | **Supplier** |
| --- | --- | --- |
| HA-hNTCP^K1-5R^ (mutant construct) | Fw:TGGTGCTATGAGAGATTCAGGACTCCCAGGGATAGAACAAGAATGATCTACACA  Rv:TGTGTATAGCATTCTTGTTCTATCCCTGGGAGTCCTGAATCTCTCATAGCACCA | Sigma-Aldrich |
| HA-hNTCP^K340R^ (mutant construct) | Fw:GCTCTGGGAAATGGCACCTACAGAGGGAGGACTGCTCC  Rv: GGAGCAGTCCTCCCCTCTGTAGGTGCCATTTCCCAG | Sigma-Aldrich |
| Seq-hNTCP 1 (mutant construct) | CATGAAGGGGGACATGAACCTC | Sigma-Aldrich |
| Seq-hNTCP 2 (mutant construct) | TGATGCCTTTTATTGGCTTT | Sigma-Aldrich |
| NTCP (qRT-PCR) | Fw:GGACATGAACCTCAGCATTGTG  Rv:GCCGTTTGGATTTGAGGACG | Sigma-Aldrich |
| 36B4 (qRT-PCR) | Fw:TCATCAACGGTACAAACGA  Rv:GAACGACTTTTCCAGTTCCG | Sigma-Aldrich |
| HBV cccDNA (qRT-PCR) | Fw:GACTCTCTCGTCCCCTTCTC  Rv:ATGGTGAGGTGAACAATGCT | Sigma-Aldrich |
| HBV DNA, rcDNA (qRT-PCR) | Fw:GTTGCCCGTTTGTCCTCTAATTC  Rv:GGAGGGATACATAGAGGTTCCTTGA | Sigma-Aldrich |
| PrP (qRT-PCR) | Fw:TGCTGGGAAGTGCCATGAG  Rv:CGGTGCATGTTTTCACGATAGTA | Sigma-Aldrich |

- 1. **Biological samples**

| **Description** | **Source** | **Identifier** |
| --- | --- | --- |
|  |  |  |

- 1. **Deposited data**

| **Name of repository** | **Identifier** | **Link** |
| --- | --- | --- |
| Mass spectrometry proteomics deposited to ProteomeXChange Consortium via PRIDE partner repository | PXD007948 | <http://www.ebi.ac.uk/pride> |

- 1. **Software**

| **Software name** | **Manufacturer** | **Version** |
| --- | --- | --- |
| LinRegPCR | Amsterdam UMC | 2013.0 |
| Graphpad Prism | Graphpad | 10 |
|  |  |  |

- 1. **Other (*e.g*. drugs, proteins, vectors etc.)**

| FLAG-tagged ubiquitin | gift of N. Zelcer |  |
| --- | --- | --- |
| NTCP constructs | Generated as described in Appelman et al. Cells. 2020 Apr 16;9(4):986. doi:10.3390/cells9040986 and Bijsmans et al. Biochem J. 2012 Feb 1;441(3):1007-15.doi:10.1042/BJ20111234. |  |
| Complete^TM^ Protease Inhibitor Cocktail | 11697498001 | Roche |
| Pierce^TM^ BCA Protein Assay Kit | 23225 | Thermo Scientific |
| HBSS phenol red free | 10-527F | Lonza |
| Dulbecco’s modified Eagle’s medium |  | Sigma-Aldrich |
| FCS |  | Gibco |
| L-glutamine |  | Lonza/Gibco |
| William’s E media |  | Gibco |
| FCS FetalClone II |  | HyClone |
| penicillin/streptomycin |  | Lonza/Gibco |
| gentamicin |  | Ratiopharm |
| Human insulin |  | Sanofi-Aventis |
| hydrocortisone |  | Pfizer |
| Fatty-acid free BSA | A6003 | Sigma-Aldrich |
| TRIzol | T92424 | Sigma |
| Chloroform | 2445 | Merck |
| Isopropyl alcohol | 1040 | Merck |
| DNase I | M6101 | Promega |
| Random Hexamer primers | SO142 | Promega |
| Revertaid transcriptase | EP0442 | Fermentas |
| Sulfo-NHS-ss-Biotin | 21331/11811205 | Fisher Scientific |
| Anti-HA agarose beads | A2095-1ML | Sigma-Aldrich |
| 2-Mercaptoethanesulfonic acid sodium salt | 19767-45-4 | Sigma-Aldrich |
| Sodium iodoacetate | 305-53-3 | Sigma-Aldrich |
| TAK-243 | HY-100487 | MedChemExpress |
| peptide N-glycosidase F | P0705S | New England Biolabs |
| [^3^H] taurocholate |  | Perkin Elmer |
| recombinant HBV encoding gaussia luciferase | Generated as described in Wing et al., Life Sci Alliance. 2019 Mar 27;2(2):e201900355.doi: 10.26508/lsa.201900355 |  |
| HBeAg Detection Reagent kit |  | Shanghai Kehua Bio-Engineering |
| NucleoSpin® Tissue DNA Isolation kit |  | Qiagen |
| Myrcludex B |  | Pepscan |
| QuikChange® Site-Directed Mutagenesis Kit |  | Agilent Technologies |
| pLenti-PGK-Hygro-DEST |  | Addgene |
| Plenti-CMV-PURO-DEST |  | Addgene |
| Gateway LR clonase II enzyme mix |  | Invitrogen |
| pENTR-D-TOPO cloning kit |  | Life Technologies |
| pVSV, pMDL and pRSV-Rev vectors | Generated as described in Dull et al. J Virol. 1998 Nov;72(11):8463-71. doi: 10.1128/JVI.72.11.8463-8471.1998. |  |
| Hygromycin |  | Merck-Millipore |
| Puromycin |  | Sigma |
| polyethyleneimine reagent |  | Brunschwig |

- 1. **Please provide the details of the corresponding methods author for the manuscript:**

| Stan van de Graaf, Amsterdam UMC, University of Amsterdam, Department of Gastroenterology and Hepatology, Tytgat Institute for Liver and Intestinal Research, Meibergdreef 69-71, Amsterdam, the Netherlands. E-mail: k.f.vandegraaf@amsterdamumc.nl |
| --- |

**2.0 Please confirm for randomised controlled trials all versions of the clinical protocol are included in the submission. These will be published online as supplementary information.**

|  |
| --- |
