# Supplementary material for: NTCP ubiquitination enables HBV infection
Source: JHEP Rep. 2025 Jul 31;7(11):101534. doi: 10.1016/j.jhepr.2025.101534 (PMC12538160; doi:10.1016/j.jhepr.2025.101534)
Supplement: Multimedia component 4 [file mmc4.pdf]

NTCP ubiquitination enables HBV infection

Authors

Monique D. Appelman, Thuc-Anh Nguyen, Andreas Oswald, …, Coen C. Paulusma, Ulrike Protzer, Stan F.J. van de Graaf

Correspondence

k.f.vandegraaf@amsterdamumc.nl (S.F.J. van de Graaf).

Graphical abstract

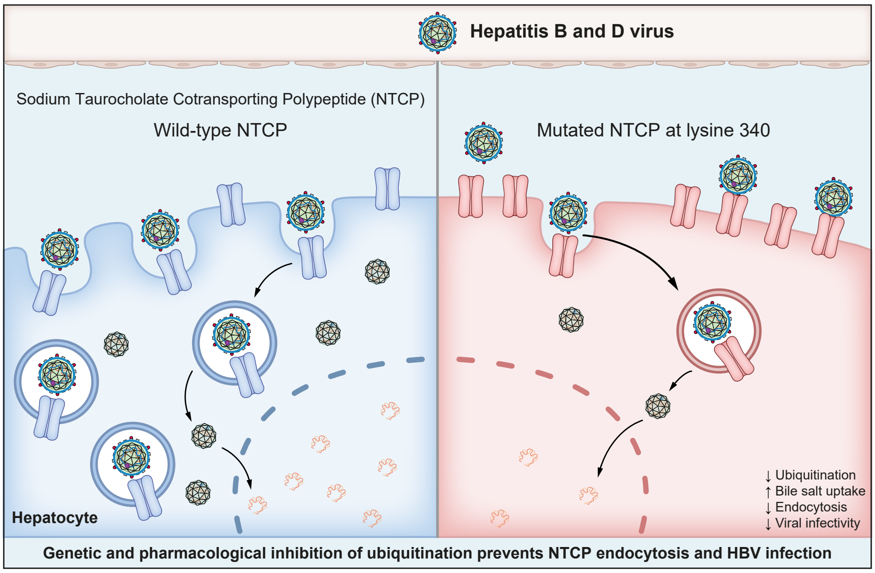

Highlights:

- K340 is likely to be the main ubiquitination target within NTCP.
- Mutating K340 to arginine increased NTCP protein levels and bile salt uptake.
- Mutating K340 to arginine reduced NTCP endocytosis and HBV infection.
- Inhibition of protein ubiquitination with TAK-243 inhibited HBV infection.
- K340 is crucial for HBV entry as a result of ubiquitin-mediated NTCP endocytosis.

Impact and implications:

This study contributes to elucidating the process of how HBV enters hepatocytes, which is largely elusive. NTCP was found not only to be required for the binding of HBV to hepatocytes, but also to have a crucial role in hepatic internalization of HBV. In addition, a K at position 340 was identified as the main ubiquitination target of NTCP; ubiquitination-mediated endocytosis of NTCP at this position is likely to be the mechanism regulating HBV internalization. Thus, interfering with NTCP ubiquitination could provide a novel means to reduce HBV infection.

https://doi.org/10.1016/j.jhepr.2025.101534
© 2025 The Authors. Published by Elsevier B.V. on behalf of European Association for the Study of the Liver (EASL). This is an open access article under the CC BY license (http://creativecommons.org/licenses/by/4.0/).

 JHEP|Reports

NTCP ubiquitination enables HBV infection

Monique D. Appelman[1,2,†], **Thuc-Anh Nguyen**[1,2,†], Andreas Oswald[3], Aaron Lucko[3], Coen C. Paulusma[1,2], Ulrike Protzer[3,4], Stan F.J. van de Graaf[1,2,*]

JHEP Reports **2025**. vol. 7 | 1–11

Check for updates

Background & Aims: The sodium taurocholate cotransporting polypeptide (NTCP), the main hepatic uptake transporter of bile salts, is the docking receptor required for the HBV/HDV entry. However, the mechanism of NTCP-dependent internalization of HBV/HDV into hepatocytes is unclear. Thus, we investigated the contribution of post-translational modification of NTCP to transporter endocytosis and HBV infection.

Methods: NTCP ubiquitination was determined by immunoprecipitation of wild-type NTCP ($NTCP^{WT}$). Lysine (K) residues in the C terminus were substituted by arginine (R) to identify ubiquitination sites. HepG2 cells overexpressing NTCP mutants were analyzed for protein levels, bile salt uptake activity, NTCP endocytosis, and HBV infectivity. The global ubiquitination inhibitor TAK-243 was used to study effects on uptake and HBV infection in $NTCP^{WT}$-HepG2 and HepaRG cells. Sample sizes in the experiments were 3–10.

Results: NTCP was found to be ubiquitinated. Compared with $NTCP^{WT}$, the $NTCP^{K340R}$ mutant showed reduced ubiquitination, indicating K340 as the main ubiquitination target. Furthermore, $NTCP^{K340R}$ had increased membrane abundance, which coincided with enhanced bile salt uptake (28.2 ± 5.3 *vs.* 74.4 ± 5.8 pmol; p <0.0001). Compared with $NTCP^{WT}$, $NTCP^{K340R}$ endocytosis was strongly impaired (100 ± 47 *vs.* 42 ± 19%; p = 0.0079), whereas HBV-derived myr-preS1 peptide binding was increased (100 ± 33 *vs.* 220 ± 98%; p <0.0001). Compared with $NTCP^{WT}$ cells, HBV DNA content was strongly reduced in $NTCP^{K340R}$ cells (52.74 ± 26.23 *vs.* 7.22 ± 3.28%; p = 0.0022). In line with this, TAK-243 reduced cellular ubiquitination levels and increased bile salt uptake (48.65 ± 2.27 *vs.* 105.8 ± 4.12 pmol; p = 0.0286), while reducing HBV DNA content in HepG2 (100 ± 44 *vs.* 18 ± 13%; p <0.0001) and HepaRG cells (100 ± 24 *vs.* 65 ± 6%; p = 0.0483).

Conclusions: K340 is essential for NTCP ubiquitination. Inhibiting ubiquitination impaired NTCP endocytosis and reduced HBV infection, confirming that NTCP-mediated endocytosis is critical for HBV hepatic entry.

© 2025 The Authors. Published by Elsevier B.V. on behalf of European Association for the Study of the Liver (EASL). This is an open access article under the CC BY license (http://creativecommons.org/licenses/by/4.0/).

Introduction

HBV infection is a major public health problem and leading cause of liver-related mortality worldwide with a death toll of over 1 million each year.[1] Chronic HBV infection leads to liver cirrhosis and hepatocellular carcinoma.[1] The most severe cases of HBV are caused by co-infection with HDV, a satellite virus that requires HBV for replication.[2] There is currently no effective medication to eliminate chronic HBV infection, but treatment focuses on suppressing viral replication and reducing the risk of complications.[1]

Recently, bulevirtide, also known as Myrcludex B, a synthetic peptide derived from the viral pre-S1 domain, was approved by the EMA for treating individuals co-infected with HBV/HDV.[3] Bulevirtide blocks HBV entry into hepatocytes by binding to the sodium taurocholate cotransporting polypeptide (NTCP), the main hepatic uptake transporter of conjugated bile salts in hepatocytes, which also inhibits its transporter activity.[3,4] As a result, the binding of this particular inhibitor induces a significant decline in viral RNA and liver inflammation in patients with HDV infection.[3]

NTCP is a transmembrane protein exclusively expressed on the basolateral membrane of hepatocytes and important in the enterohepatic circulation of bile salts as the main uptake transporter of conjugated bile salts from the portal blood into the liver.[5] A certain genomic mutation, p.Ser267Phe (rs2296651), which has an allele frequency of 8–12% in Southern Chinese and 11% in Vietnamese populations, is associated with decreased susceptibility to HBV/HDV infection.[6–9] In homozygous individuals, the S267F variation was reported to be associated with elevated plasma bile salt levels as a result of reduced NTCP function, which, however, did not result in any medical consequences in this population.[6] Furthermore, NTCP expression levels have been shown to be positively correlated with HBV DNA levels in individuals with chronic HBV.[10] However, expression of NTCP was decreased in more severe HBV cases developing significant liver fibrosis and tissue damage.[10] Thus, it is clear that NTCP has a crucial

* Corresponding author. Address: Tytgat Institute for Liver and Intestinal Research, Academic Medical Center, Meibergdreef 69-71, 1105 BK Amsterdam, The Netherlands. Tel.: +31-(0)20-5663828
E-mail address: k.f.vandegraaf@amsterdamumc.nl (S.F.J. van de Graaf).
† These authors contributed equally.
https://doi.org/10.1016/j.jhepr.2025.101534

role in HBV/HDV cellular entry and infection. However, the underlying mechanisms and pathways that regulate the entry of HBV into hepatocytes remain largely elusive.

Ubiquitination is the covalent attachment of a small (76-amino acid) protein to an acceptor lysine (K) residue in target proteins, and regulates various cellular processes, including protein trafficking, endocytosis, and degradation.[11,12] Both inhibition of the proteasome by MG-132 or lactacystin[13] and inhibition of lysosomes using bafilomycin A1[14] increased intracellular NTCP levels, indicating the involvement of ubiquitin-mediated degradation of NTCP. Poly-ubiquitination of NTCP in aggresomes was also detected, suggesting endoplasmic reticulum (ER)-associated degradation of NTCP upon overexpression and in patients with cholestasis. Whether ubiquitination has a role at more physiological levels of NTCP expression and makes a possible contribution to NTCP endocytosis and/or cellular HBV entry remains unknown. Here, we investigated the post-translational modification of NTCP by ubiquitination as a potential signal for NTCP endocytosis and as a mechanism of NTCP-mediated HBV internalization.

Materials and methods

Cell lines and culture

Human hepatocellular carcinoma cells (HepG2), human osteosarcoma cells (U2OS), human embryonic kidney cells (HEK293T) (all from ATCC, Manassas, VA, USA) were grown in DMEM (Sigma-Aldrich, Grand Island, NY, USA), supplemented with 10% fetal calf serum (FCS; Gibco, Carlsbad, CA, USA), 1% L-glutamine (Lonza, Basel, Switzerland), and 1% penicillin/streptomycin (Lonza) (Supplementary CTAT Table). Cell lines were passaged twice a week at a confluence of 80% and incubated in a humidified atmosphere of 37 °C and 5% CO_2. HepaRG cells were cultured with William's E media (Gibco, Grand Island, NY, USA) supplemented with 10% FCS FetalClone II (HyClone, Little Chalfont, UK), 20 mM L-glutamine (Gibco), 50 U/ml penicillin/streptomycin (Gibco), 80 µg/ml gentamicin (Ratiopharm, Ulm, Germany), 0.023 IE/ml human insulin (Sanofi-Aventis, Paris, France), and 4.7 µg/ml hydrocortisone (Pfizer, Carlisle, PA, USA) as described previously.[15] HepaRG differentiation was also performed as described previously.[15,16]

Generation of NTCP mutant constructs

Mutations in the NTCP construct were generated using the QuikChange® Site-Directed Mutagenesis Kit (Agilent Technologies, Santa Clara, CA, USA). NTCP open reading frames were cloned into the vector pLenti-PGK-Hygro-DEST (Addgene, Watertown, MA, USA) or the Plenti-CMV-PURO-DEST using the Gateway LR clonase II enzyme mix (Invitrogen, Waltham, MA, USA) after initial cloning into pENTR-D-TOPO according to the manufacturer's instructions (Life Technologies, Waltham, MA, USA). All constructs were sequence verified. Primers used to generate the constructs can be found in Table S1 and in the Supplementary CTAT Table. These constructs were used for both transient transfections and the generation of stable cell lines.

Generation of cell lines

U2OS cells and HepaRG cells stably expressing human NTCP were generated as previously described.[16,17] For the generation of stable expressing NTCP cell lines, HEK293T cells were seeded in 100-mm plates, 24 h before transfection with third-generation virus plasmids pVSVg, pMDL, and pRSV-Rev vectors and one of the NTCP constructs. Medium from the HEK293T cells was harvested and added to HepG2 cells or U2OS cells for 6 h followed by refreshing of the medium. After 48 h, the infected cells were selected using Hygromycin (50 µg/ml, Merck-Millipore, Burlington, MA, USA) or Puromycin (2.5 µg/ml, Sigma-Aldrich). Transient transfections with FLAG-tagged ubiquitin (gift from N. Zelcer[18]) and NTCP constructs were performed using polyethyleneimine (Brunschwig, Basel, Switzerland) as previously described.[19]

NTCP plasma membrane expression and internalization assay

NTCP plasma membranes were determined by cell surface biotinylation as previously described.[14,20,21] For the internalization assays, cells were incubated with 1 mg/ml Sulfo–NHS–ss-Biotin for 1 h at 4 °C. After unreacted biotin was quenched, cells were incubated at 37 °C for 1.5 h to allow internalization of biotinylated protein. Internalization was ceased by cooling cells to 4 °C. To measure NTCP internalization, biotin that remained at the cell surface was removed by incubation with fresh 100 mM 2-mercaptoethanesulfonic acid sodium salt (MESNA, Sigma-Aldrich) in 100 mM NaCl, 1 mM EDTA, 50 mM Tris-HCl, pH 8.6, 0.2% (w/v) bovine serum albumin three times for 20 min at 4 °C. Subsequently, MESNA was quenched for 10 min with 120 mM sodium iodoacetate (Sigma-Aldrich) in PBS-CM. Cells were washed with PBS-CM and PBS and lysed in 150 mM NaCl, 5 mM EDTA, 50 mM Tris-HCl, pH 7.5, 1% (v/v) Nonidet P-40, supplemented with protein inhibitors (Roche, Basel, Switzerland) at 4 °C. Lysate was centrifuged, and the supernatant was added to prewashed neutravidin beads. Pulldown was performed overnight at 4 °C followed by washing with lysis buffer. The proteins were eluted in Laemmli sample buffer containing 0.1 M DTT, incubated at room temperature for 15 min, and then subjected to immunoblotting as described below.

TAK-243 treatment

$NTCP^{WT}$ HepG2 and HepaRG cells (only HBV infection) were plated for bile salt uptake or HBV infection assays. After 48 h, cells were treated with TAK-243 (MedChemExpress, Monmouth Junction, NJ, USA) at selected concentrations for 3 h (HBV infection) or overnight (bile salt uptake assay).

PNGase treatment

Lysates of $NTCP^{WT}$ HepG2 (60 µg) were treated with peptide N-glycosidase F (PNGase F) for 2 h at 37 °C (500 units) according to the manufacturer's instructions (New England Biolabs, Ipswich, MA, USA) before immunoblotting, as described below.

Immunoblot analyses

Proteins were separated on SDS-PAGE gels and transferred to PVDF membranes using the Bio-Rad system. After transfer, membranes were blocked in 5% milk/TBS-Tween (TBS-T) for 1 h and then incubated with primary antibodies for either 2 h at room temperature or overnight at 4 °C. After three washing

steps with TBS-T, membranes were incubated with horseradish peroxidase-conjugated secondary antibodies (either goat anti-mouse or goat anti-rabbit (1:10,000)). Following antibody incubations and three additional washing steps, protein bands were visualized using Lumi-Light PLUS Western Blotting Substrate (Roche), and proteins were detected by chemiluminescence. Primary antibodies included anti-HA HRP (H6533, Sigma-Aldrich), rabbit anti-FLAG (F7425, Sigma-Aldrich), mouse anti-FLAG (F1804, Sigma-Aldrich), anti-ubiquitin-HRP (BML-PW0150, ENZO, Farmingdale, NY, USA), rabbit anti-ATP1A1 (gift from J. Koenderink[22]), and rabbit anti-GAPDH (CST 2118S, Cell Signaling, Danvers, MA, USA). ATP1A1 served as the loading control in the immunoblot experiments. For secondary antibodies, anti-mouse-HRP (P0447, DAKO, Glostrup, Denmark) and anti-rabbit-HRP (31460, Thermo Fisher Scientific) were used (Supplementary CTAT Table).

Immunoprecipitations

Cells were grown in a 100-mm culture dish until 80% confluence. After washing with PBS, cells were lysed in RIPA buffer (150 mM NaCl, 50 mM Tris pH 7.4, 0.1% SDS, 1% Nonidet P40, 1 mM EDTA) supplemented with protein inhibitors (Roche). Equal protein amounts were incubated and FLAG-NTCP or HA-NTCP precipitation was performed. For HA-NTCP, precipitation was performed by incubating the lysates with monoclonal anti-HA antibody immobilized on agarose beads (9568, Sigma-Aldrich) for 16 h at 4 °C. For FLAG-NTCP, lysates were incubated with anti-FLAG antibodies (F1804, Sigma-Aldrich) overnight at 4 °C and, subsequently, protein agarose beads were added for another 2 h at 4 °C. After three washing steps with RIPA buffer, samples were analyzed by immunoblotting, as described above.

RNA isolation, cDNA synthesis, and qPCR

RNA was isolated from cells as described previously.[20,23] The primers (Sigma-Aldrich) used are listed in Table S2 and the Supplementary CTAT Table. Relative expression was determined by qPCR, and expression levels were normalized to two reference genes, *36B4* and *HRPT*.

Bile salt uptake assay

Bile salt uptake activity was measured in cells as described previously using [^{3}H] taurocholate (TCA; Perkin Elmer).[14] Briefly, cells were grown in 24-well plates until 80% confluence. Cells were washed twice with warm uptake buffer (5 mM KCl, 1.1 mM KH_2PO_4, 1 mM $MgCl_2$, 1.8 mM $CaCl_2$, 10 mM D-glucose, 10 mM Hepes, and 136 mM NaCl). Uptake activity was examined by incubation with 20 μM TCA supplemented with 0.25 μC [^{3}H] TCA in uptake buffer at 37 °C for 2 min. Finally, cells were washed four times in ice-cold PBS and lysed in 0.05% SDS. Accumulation of radiolabeled substrates was determined by scintillation counting.

HBV binding to NTCP

HBV binding to NTCP was determined by Myrcludex-FITC labeling as previously described.[14,20]

Determination of HBV/HDV infection

HepG2 cells expressing NTCP constructs were cultured in DMEM/F-12 medium containing 10% FCS, 1 mM sodium pyruvate, 1% nonessential amino acids, 1% L-glutamate, and 2 mM HEPES. HepaRG cells were cultured and differentiated as described above. Cells were seeded in a 24-well plate at 90% confluency with 2% DMSO in the medium. After 48 h, they were infected with HBV at a multiplicity of infection (MOI) of 200 or 500 DNA-containing, enveloped viral particles (vp)/cell in a medium containing 2% DMSO and 4% polyethylene glycol (PEG) 6000. Negative control cells were pretreated with 200 nM Myrcludex B for 1 h before infection.

To assess the effects of global inhibition of ubiquitination, cells were treated with TAK-243 at 0.25, 0.5, or 10 μM for 3 h before HBV infection and washed three times with PBS. These cells were then infected with 200 MOI vp/cell of a recombinant HBV encoding *Gaussia* luciferase under the control of a transthyretin promoter generated as previously described.[24] Then, 24 h post infection, cells were washed and supplemented with fresh medium without PEG6000 and the cells and cellular supernatant were then harvested. Total DNA was extracted from the supernatant and cells using the NucleoSpin® Tissue DNA Isolation kit (Qiagen, Hilden, Germany). Total HBV DNA was quantified using qPCR as previously described.[25] HBV covalently closed circular (ccc) DNA was determined by qPCR following T5 exonuclease pretreatment, and the expression levels were normalized to the major prion protein (PrP) level. HBeAg was quantified in the supernatant by ELISA using the HBeAg Detection Reagent kit (Shanghai Kehua Bio-Engineering, Shanghai, China). *Gaussia* luciferase activity was measured by adding 50 μl of supernatant to a white 96-well plate and adding 100 μl of PBS-T (0.1% Tween 20) with 1 μM coelenterazine H before measuring luminescence with a Tecan Infinite 200 plate reader.[24]

Statistical analyses

Data are provided as the mean ± SD. Differences between two groups were analyzed using the Mann-Whitney *U* test. One-way ANOVA with Dunnet or Bonferroni *post hoc* analysis was used for multiple group comparisons. Statistical significance was considered at $p < 0.05$. Calculations and graphs were generated using GraphPad Prism 10.2.0 (GraphPad Software Inc., La Jolla, CA, USA).

Results

NTCP can be ubiquitinated

Given that ubiquitin attachment to membrane proteins on the plasma membrane targets them for internalization, we investigated whether NTCP can be ubiquitinated. To investigate this, U2OS cells with stable expression of HA-tagged human NTCP were generated and transfected with FLAG-tagged ubiquitin. Precipitation of HA-NTCP resulted in co-precipitation of FLAG-ubiquitin (Fig. 1A). The omission of NTCP prevented the precipitation of ubiquitin, demonstrating the specificity of the co-immunoprecipitation.

To exclude that the transiently overexpressed ubiquitin affected these results, the experiment was repeated without overexpression of ubiquitin. Precipitation of HA-NTCP resulted in co-precipitation of endogenous ubiquitin, which implies that

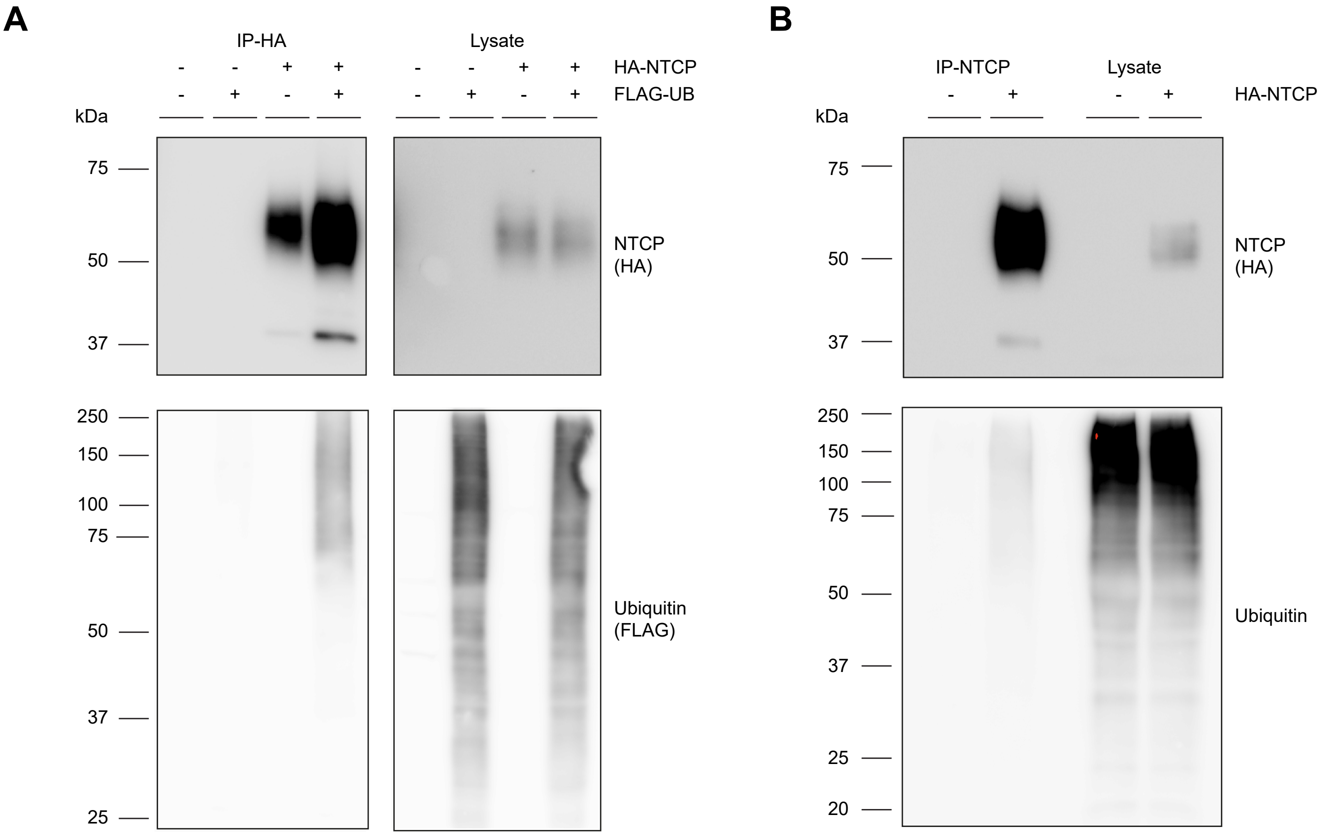

Fig. 1. NTCP physically interacts with ubiquitin. (A) FLAG-ubiquitin coprecipitates with HA-NTCP in U2OS cells stably expressing HA-tagged NTCP and transiently overexpressing FLAG-tagged ubiquitin. (B) Endogenous ubiquitin coprecipitates with HA-NTCP in U2OS cells. (A,B) NTCP was immunoprecipitated with anti-HA antibodies and immunoblotted with anti-HA-HRP and anti-ubiquitin-HRP. Shown is a representative experiment of n = 2–3 independent experiments. Full blots are shown in Fig. S1. HRP, horseradish peroxidase; NTCP, sodium taurocholate cotransporting polypeptide.

NTCP can be ubiquitinated (Fig. 1B). Furthermore, ubiquitin precipitated with NTCP was detected as multiple bands between 50 and 250 kDa, which suggests that NTCP can be both mono- and poly-ubiquitinated (Fig. 1A,B).

K340R mutation results in increased NTCP levels and bile salt uptake but is less ubiquitinated

Identifying the main target site for ubiquitination within NTCP can provide a means to modify and inhibit NTCP ubiquitination to understand its role in endocytosis and viral entry. The target sites for ubiquitination are K residues located in the cytoplasmic region of a transmembrane protein, given that ubiquitination occurs intracellularly. In 2022, the crystal structure of NTCP was resolved, demonstrating that it contains nine transmembrane domains and an intracellularly localized C terminus (Fig. 2A).[26] To assess whether ubiquitination of human NTCP is important for its internalization and, hence, HBV entry, we focused on the six K residues in the C terminus. Four K residues, at positions 309, 311, 314, and 318 are conserved in both HBV-susceptible (human, chimpanzee, and bonobo) and non-susceptible (mouse and rat) species,[27–29] whereas K residues at positions 316 and 340 are not conserved (Fig. 2B). To investigate whether K residues are relevant for NTCP activity and internalization, we substituted all residues with an arginine (R) residue by site-directed mutagenesis, and expressed these mutants in HepG2 cells: wild-type NTCP ($NTCP^{WT}$), $NTCP^{K1\text{-}5R}$ in which five K residues were mutated (K309R, K311R, K314R, K316R, and K318R), $NTCP^{K1\text{-}6R}$ in which six K residues were mutated (K309R, K311R, K314R, K316R, K318R, and K340R), and $NTCP^{K340R}$ in which only K residue K340 was mutated. Despite comparable *NTCP* mRNA expression for all mutant constructs (Fig. 2C), NTCP protein levels in whole-cell lysates were strongly elevated only for the $NTCP^{K1\text{-}6R}$ and $NTCP^{K340R}$-expressing lines (Fig. 2D, lysate). Interestingly, the plasma membrane abundance of these two mutants was strongly enhanced, as determined by cell surface biotinylation (Fig. 2D, eluate). The increased plasma membrane abundance coincided with approximately threefold elevated TCA uptake by these two NTCP mutant cell lines compared with $NTCP^{WT}$ and $NTCP^{K1\text{-}5R}$ (Fig. 2E). Altogether, these data indicate that mutation of K340 has the strongest phenotype in relation to NTCP plasma membrane abundance and transport activity.

Next, we assessed whether the general inhibition of protein ubiquitination mirrored the increased uptake activity of $NTCP^{K340R}$. To this end, HepG2-$NTCP^{WT}$ cells were treated with TAK-243, an inhibitor of the ubiquitin-activating enzyme. Overnight treatment with TAK-243 at 0.1 μM reduced ubiquitination (Fig. S3A) and, as we observed for $NTCP^{K340R}$, strongly increased TCA uptake (Fig. 2F). To investigate whether K340 is the target for ubiquitination, we precipitated

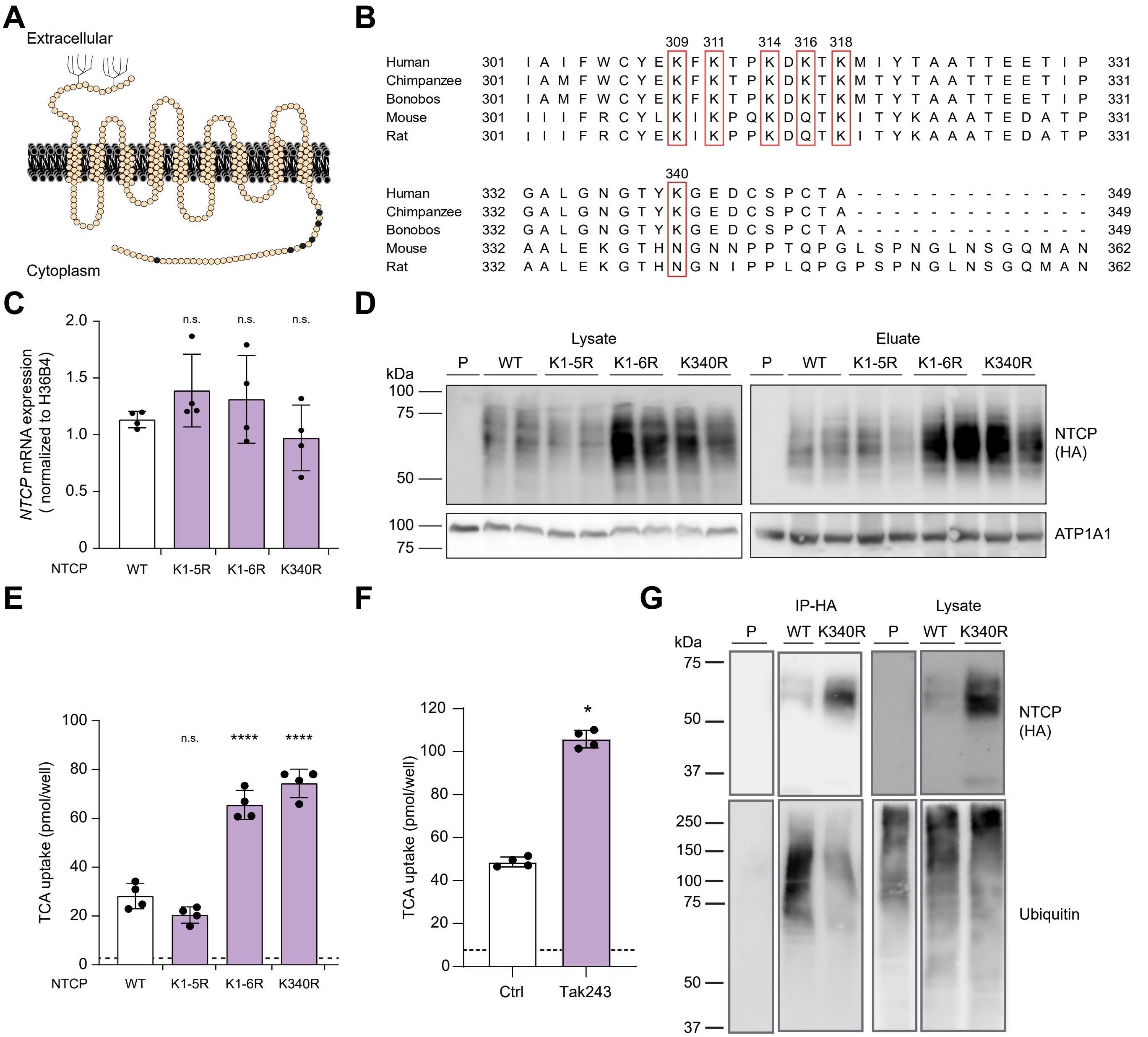

Fig. 2. NTCP^{K340R} associates with increased NTCP plasma membrane levels and uptake activity and K340 is the main target for ubiquitination. (A) Schematic topological model of human NTCP highlighting the K residues in the C terminus (black). (B) Multiple sequence alignment of NTCP C-terminal sequences from different species in which the human conserved K residues are boxed. (C–E) Experiments performed in HepG2 cells stably expressing NTCP^{WT}, NTCP^{K1-5R}, NTCP^{K1-6R}, or NTCP^{K340R}. (C) *NTCP* mRNA expression. (D) Representative immunoblot from surface biotinylation experiment showing NTCP protein levels in total lysate (left) and at the plasma membrane (right) (n = 3). (E) TCA uptake. (F) TCA uptake in HepG2 cells stably expressing NTCP^{WT} treated overnight with 0.1 μM TAK-243. (G) Immunoprecipitation of HA-hNTCP in U2OS cells stably expressing HA-NTCP^{WT} or HA-NTCP^{K340R}. Full blots are shown in Figs. S2 and S4. Data are means ± SD, analyzed with one-way ANOVA followed by Dunnet's test (C,E) and Mann-Whitney *U* test (F); ns, not significant, **p* <0.05, *****p* <0.0001. K, lysine; NTCP, sodium taurocholate cotransporting peptide; P, parental cells without HA-NTCP expression; TCA, taurocholate; WT, wild type.

HA-NTCP from U2OS cells overexpressing either HA-NTCP^{WT} or HA-NTCP^{K340R} and compared the ubiquitination of the precipitated protein. The precipitated ubiquitin level was strongly reduced in NTCP^{K340R} compared with NTCP^{WT}, even though NTCP^{K340R} expression levels were higher (Fig. 2G). These data suggest that K340 is the main target site for ubiquitination within NTCP.

Given that we previously showed that the *N*-glycosylation of NTCP is essential for the recruitment of NTCP to the plasma membrane and subsequently HBV entry by NTCP, we examined glycosylation of NTCP^{WT} and NTCP^{K340R}.[14] Cells were treated with PNGase F, which cleaves *N*-linked glycan chains from glycoproteins. The K340R mutation did not affect the glycosylation status of NTCP, indicating that the increased

plasma membrane abundance of $NTCP^{K340R}$ was not caused by impaired glycosylation of the protein (Fig. S5A).

Ubiquitination of K340 is required for endocytosis of NTCP

The increased plasma membrane abundance of $NTCP^{K340R}$ could result from impaired endocytosis caused by reduced ubiquitination. To test this hypothesis, endocytosis rates of $NTCP^{WT}$ and $NTCP^{K340R}$ were evaluated in HepG2 cells. We used a pulse-chase approach (Fig. 3A) in which membrane-associated proteins, including NTCP, were labeled with biotin at 4 °C (fraction: total plasma membrane NTCP) and chased at 37 °C to allow internalization (fraction: internalized NTCP). The endocytosed fraction was determined by dividing the fraction of internalized NTCP by the fraction of plasma membrane NTCP, both corrected for background. NTCP expression in all the aforementioned conditions was normalized to the household protein ATP1A1. Omission of biotin prevented the precipitation of NTCP (Fig. S6, lanes 1–4), whereas, in whole-cell lysate, NTCP was detected (Fig. S6, lanes 5–8). Incubations performed at 4 °C, which depicted maximal membrane-associated (biotinylated) NTCP signal (Fig. 3B, lanes 1–6) confirmed that the $NTCP^{K340R}$ had strongly increased protein levels compared with $NTCP^{WT}$. Subsequent chase of the cells for 1 h at 37 °C, followed by MESNA treatment to clear residual membrane-associated biotinylated NTCP, showed the internalized fraction of biotinylated NTCP (Fig. 3B, lanes 7–12). The internalized fraction of $NTCP^{K340R}$ was lower than that of $NTCP^{WT}$ (Fig. 3C). These experiments demonstrate that K340 has a crucial role in NTCP endocytosis, given that its mutation leads to decreased endocytosis.

The $NTCP^{K340R}$ mutant and inhibition of ubiquitination markedly reduce HBV infection

We investigated whether the endocytosis-defective $NTCP^{K340R}$ mutant expressed in HepG2 cells affected HBV infection. First, we quantified the binding of FITC-labeled Myrcludex B to $NTCP^{WT}$ and $NTCP^{K340R}$ in HepG2 cells. Myrcludex B is a myristoylated peptide based on the pre-S1 domain of the HBV-L protein that specifically interacts with NTCP at positions 157–165.[14,30–32] Therefore, this peptide indicates the HBV-binding capacity of NTCP. Myrcludex B-FITC intensity was strongly increased in HepG2-$NTCP^{K340R}$ cells (Fig. 4A), underscoring the higher plasma membrane abundance of the K340R mutant with the preserved PreS1 binding of $NTCP^{K340R}$ and indicating the higher HBV binding capacity of these cells. Next, HepG2-$NTCP^{WT}$ and $NTCP^{K340R}$ cells were incubated with HBV at MOI 500 and 200 vp/cell. At both MOIs, the HBV markers HBsAg (Fig. 4B), cccDNA (Fig. 4C), and secreted HBV DNA (Fig. 4D) were reduced by ~80% in HepG2-$NTCP^{K340R}$ cells compared with HepG2-$NTCP^{WT}$ cells. Myrcludex B, which blocks NTCP-mediated HBV entry, and heparin, which blocks the binding of HBV to highly sulfated heparan sulfate proteoglycan, were used as controls. Both treatments almost completely inhibited HBV infection, illustrating its NTCP dependency and the specificity of HBV infection. Altogether, these data demonstrate that the $NTCP^{K340R}$ still supports HBV infection, but with much lower infectivity compared with $NTCP^{WT}$, despite increased total cellular binding of PreS1.

Finally, we verified whether decreased general cellular ubiquitination affects HBV infection. HepG2 cells overexpressing $NTCP^{WT}$ were treated with TAK-243 at 0.25, 0.5, and 10 μM for 3 h. Next, cells were infected with recombinant HBV encoding *Gaussia* luciferase (rHBV-*Gaussia*) at 200 MOI vp/cell. TAK-243 treatment for 3 h reduced ubiquitination in $NTCP^{WT}$ HepG2 cells (Fig. S3B). Correspondingly, HBV markers, including luciferase expression, secreted HBV DNA, and cccDNA, were significantly reduced (Fig. 5A–C). To further verify this observation in another HBV infection model, this experiment was also performed in differentiated HepaRG cells overexpressing $NTCP^{WT}$. TAK-243 treatment showed a concentration-dependent reduction in rHBV-Gaussia activity, HBV DNA, and cccDNA (Fig. 5D,E). Altogether, these data underscore an essential role of NTCP ubiquitination at the K340 residue in HBV infection.

Discussion

In this study, we present evidence that ubiquitination of K340 in the C terminus of NTCP is crucial for NTCP endocytosis and for HBV entry and infection. First, we showed that NTCP is ubiquitinated and that the lysine residue at position 340 is the main ubiquitination target. Second, we demonstrated that mutation of this lysine to arginine (K340R) resulted in increased NTCP abundance on the plasma membrane and increased bile salt uptake. Third, the $NTCP^{K340R}$ mutant showed impaired endocytosis efficiency. In addition, we demonstrated that, despite higher Myrcludex/PreS1 binding to the HepG2-$NTCP^{K340R}$ mutant cells, these cells had a strongly reduced HBV infection capacity compared with $NTCP^{WT}$. Lastly, we demonstrated that the ubiquitination inhibitor TAK-243 reduced HBV infection in both NTCP-expressing HepG2 and HepaRG cells. Altogether, our data indicate that K340 in NTCP has a crucial role in HBV entry via ubiquitination-mediated endocytosis of NTCP.

The identification of K340 as a residue in NTCP that is affecting ubiquitination and endocytosis adds to a compact list of amino acid residues that are either pivotal in HBV infection or separate the two 'roles' of NTCP: bile salt uptake and HBV docking. This list now includes S267, a pore-residing residue crucial for bile salt and HBV binding,[26] the polymorphism G158R, and variants at the Y146 residue that block HBV binding and subsequent infection, but not bile salt transport.[29,33,34] In addition, amino acid residues 157–165 and 84–87 are essential for viral infection.[4,35] Interestingly, these are present in distinct regions of NTCP compared with the K340 position, which is in the C terminus, pointing at highly divergent mechanisms that could be exploited to interfere with HBV infection therapeutically. Yan *et al.* demonstrated that HDV delta antigen binding was reduced when the human NTCP tail was exchanged for the mouse NTCP tail,[35] although the underlying mechanism was not further investigated. The K340R mutation is unlikely to affect hNTCP oligomerization, which modulates HBV infection, because this process is independent of the C terminus.[17] The NTCP glycosylation status was unaffected in the K340R mutant, excluding another factor that modulates NTCP cell surface abundance and HBV infection.[14] Therefore, we conclude that reduced NTCP endocytosis resulting from a lack of ubiquitination explains the increased bile salt uptake and reduced HBV infection.

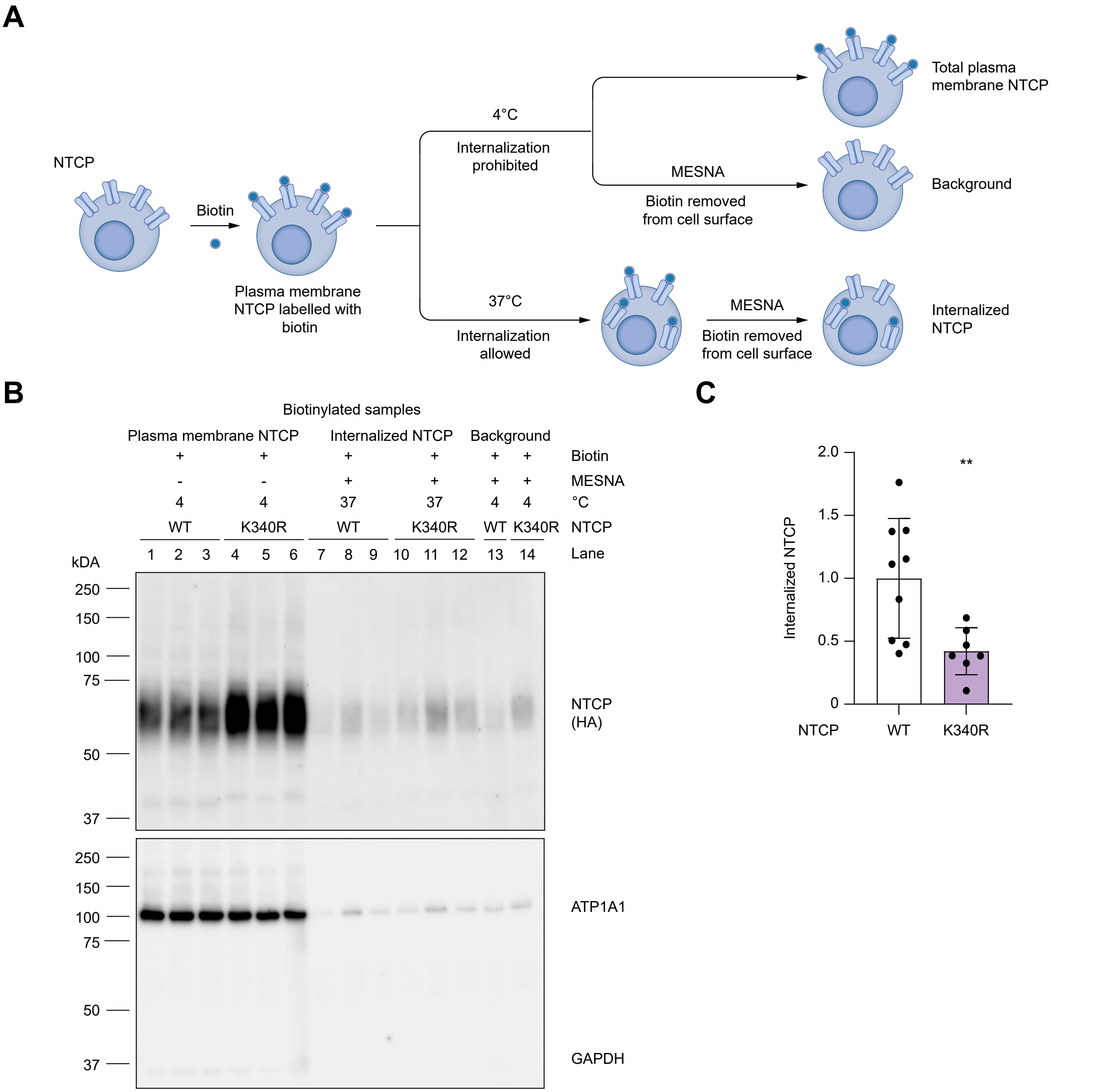

Fig. 3. NTCP^{K340R} has reduced endocytosis. (A) Schematic overview of the endocytosis assay. Endocytosis was examined using biotin pulse-chase labeling of plasma membrane proteins in which total plasma membrane NTCP was determined from biotinylated cells kept at 4 °C and endocytosis by subsequently incubating the cells at 37 °C for 1.5 h followed by MESNA treatment to remove biotin remaining on the plasma membrane. (B) Representative blot of endocytosis assay of NTCP^{WT} and NTCP^{K340R} in HepG2 cells; n = 3 independent experiments. Full blots are shown in Fig. S7. (C) Quantification of endocytosis fractions. Data are means ± SD, analyzed with Mann-Whitney *U* test; ***p* <0.001. K, lysine; MESNA, 2-mercaptoethanesulfonic acid sodium salt; NTCP, sodium taurocholate cotransporting peptide; WT, wild type.

The role of ubiquitin as a signal for internalization has been described for various membrane proteins, including transporters for dopamine,[36–39] glutamate,[40] and glycine.[41,42] For these transporters, it was shown that ubiquitination is essential for protein kinase C (PKC)-induced, clathrin-dependent endocytosis, which coincided with reduced membrane-associated transport activity. For example, the transport activity of the glycine transporter GLYT2 was reduced upon PKC activation (and consequent endocytosis). However, for cells expressing the GLYT2^{K791R} in which the ubiquitin target lysine residue 791 was replaced by an arginine (K791R), transport activity and endocytosis were unaffected upon PKC activation.[41] Ubiquitination of the essential lysine residue precedes PKC-induced clathrin-mediated internalization of these

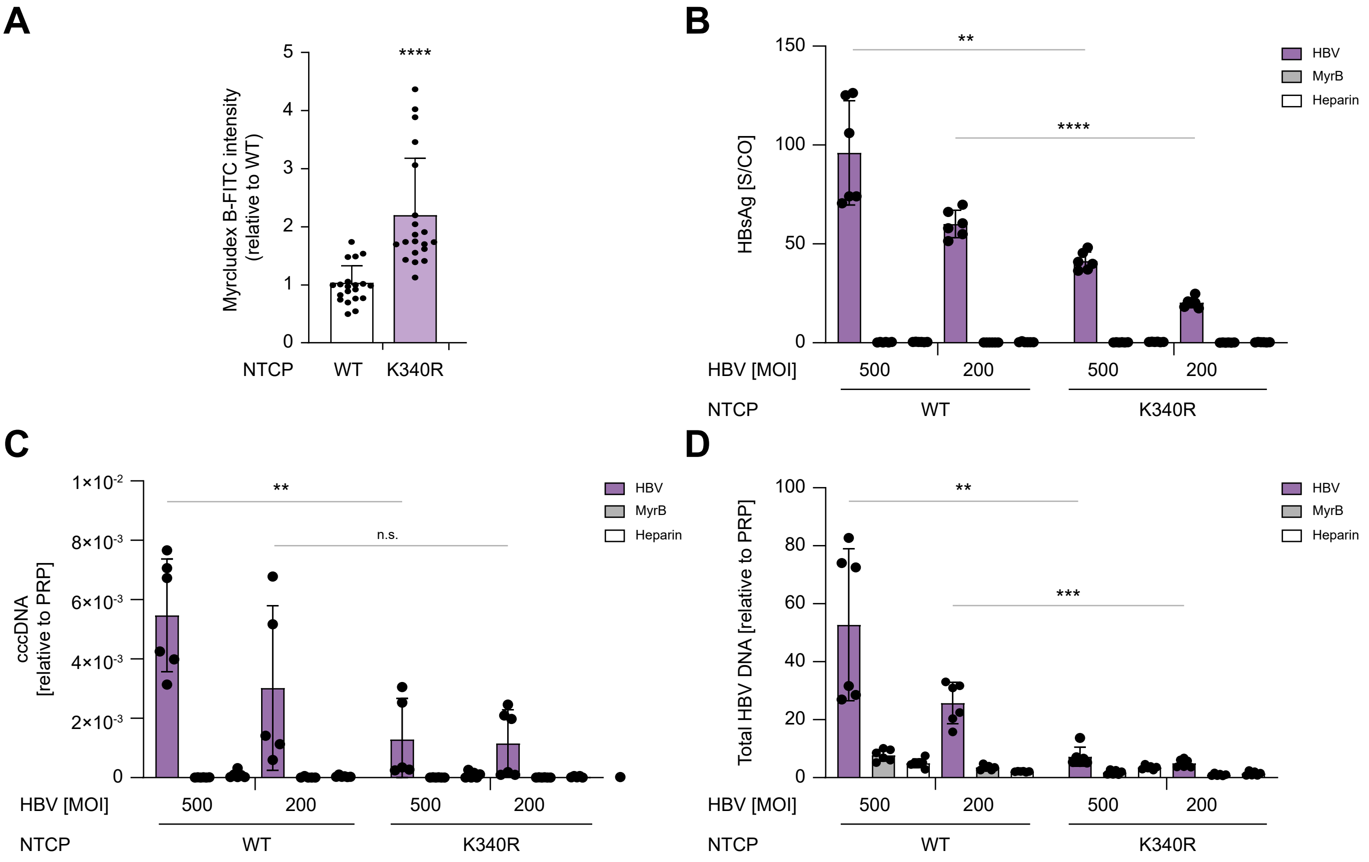

Fig. 4. NTCP^{K340R} cells show decreased HBV infection. (A) Quantification of Myrcludex B-FITC binding to HepG2-NTCP^{WT} or -NTCP^{K340R} cells. Data are shown relative to NTCP^{WT} and combined from three independent experiments. (B–D) Markers of HBV infection in HepG2-NTCP^{WT} and HepG2-NTCP^{K340R} cells infected with HBV at MOI 500 and 200. HBV infection was inhibited by Myrcludex B and heparin. HBsAg (B) results were normalized S/Co. Other markers include cccDNA (C) and total HBV DNA (D). Data are means ± SD, analyzed with Mann-Whitney *U* test; ns, not significant, $^{*}p$ <0.05, $^{**}p$ <0.01, $^{***}p$ <0.001, $^{****}p$ <0.0001. cccDNA, covalently closed circular DNA; K, lysine; MOI, multiplicity of infection; NTCP, sodium taurocholate cotransporting peptide; S/Co, ratio of sample signal to internal cut-off signal; WT, wild type.

membrane proteins. Using flow cytometry and immunofluorescent analyses, Stross *et al.*[43,44] previously showed that endocytosis of NTCP also relies on activation of PKC and clathrin. Importantly, several studies also reported that HBV entry in hepatocytes relies on clathrin-mediated endocytosis of NTCP. Using HepG2-NTCP cells, Herrscher *et al.*[45] showed that HBV entry depends on clathrin-mediated endocytosis because HBV internalization was inhibited by knocking down clathrin heavy chain, dynamin-2, or clathrin adaptor protein AP-2, but not on micropinocytosis and caveolin-mediated endocytosis. Similarly, Huang *et al.*[46] showed that HBV internalization depends on clathrin-mediated endocytosis in immortalized human primary hepatocytes. In addition, Iwamoto *et al.*[47] demonstrated that NTCP-mediated HBV internalization was dependent on the physical interaction with the epidermal growth factor receptor (EGFR) and on the endosomal sorting machinery of the EGFR. However, we could not confirm this interaction in hNTCP-overexpressing HepG2 cells.[20,23]

Our study adds another layer of regulation to NTCP-mediated HBV internalization. We showed that NTCP is ubiquitinated at K340 in the C terminus, and has an essential role in the endocytosis of NTCP. Despite strongly elevated NTCP^{K340R} membrane levels and concomitant elevated Mycrcludex B (HBV) binding, the HBV infection efficiency was reduced by ~80%, indicating that NTCP-mediated HBV internalization was strongly impaired when ubiquitination at K340 was blocked. HBV is not the only virus that requires ubiquitination of the host docking protein for virus endocytosis. Dengue virus (DENV) requires ubiquitination of two cytosolic lysine residues in the phosphatidylserine receptor TIM-1 to be endocytosed into the host cell.[48] Lysine-to-arginine mutation of both residues at positions 338 and 346 (KKRR) resulted in reduced ubiquitination and a concomitant ~75% reduction in DENV-infected cells. However, also in this study, the authors identified a single lysine residue (*i.e.* K346) to be essential for TIM-1-mediated DENV internalization. This study provides a strong link between the requirement of lysine ubiquitination and viral endocytosis.

In conclusion, we show that NTCP-mediated internalization of HBV is strongly dependent on the ubiquitination of K340. Ubiquitination of this specific residue drives NTCP endocytosis and is pivotal for efficient HBV infection. Thus, pharmacological inhibition of ubiquitin-dependent endocytosis of NTCP provides a novel strategy to block HBV entry and infection.

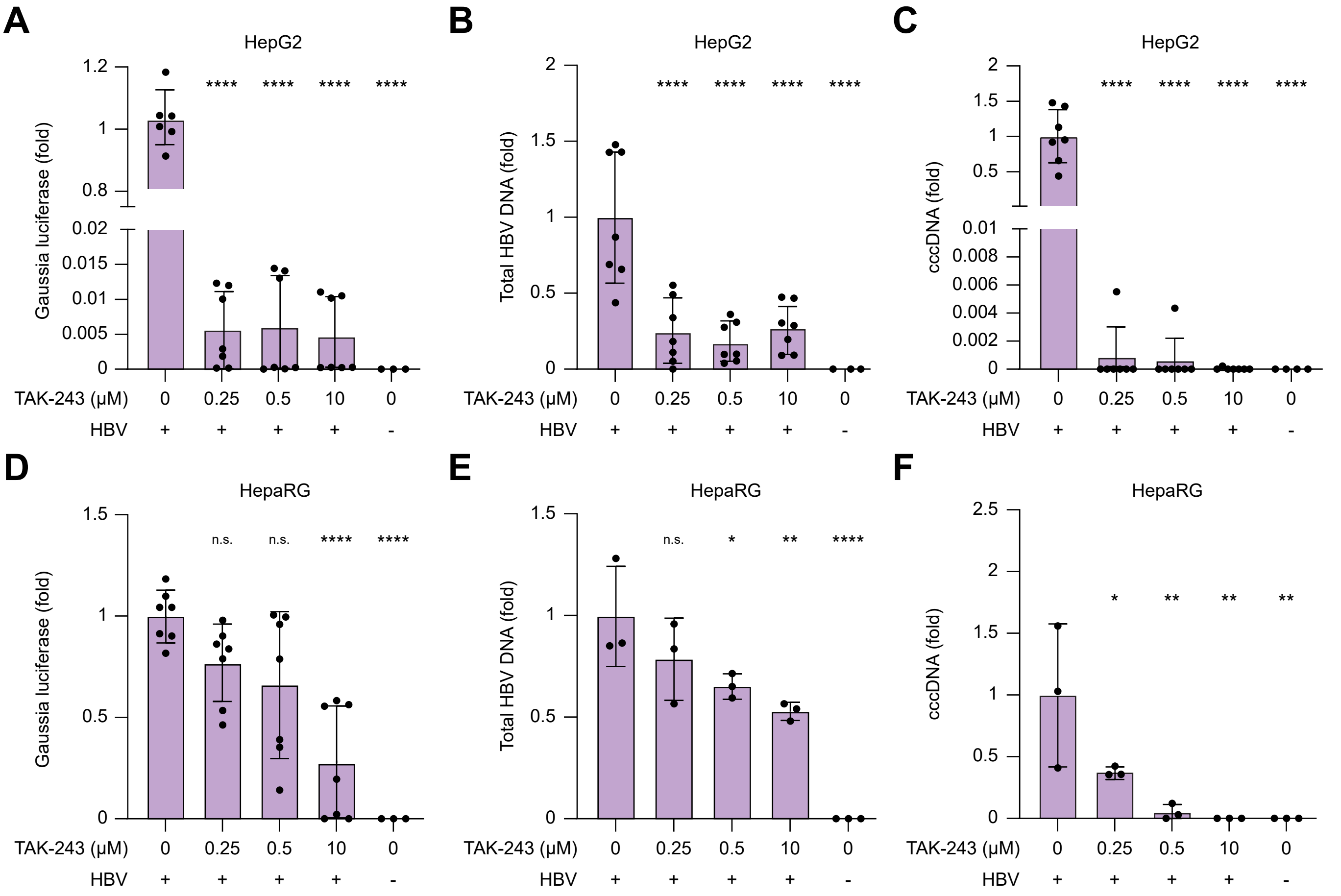

Fig. 5. Ubiquitination inhibition reduces HBV infection. HepG2-overexpressing $NTCP^{WT}$ (A–C) and HepaRG-overexpressing $NTCP^{WT}$ cells (D–F) treated with TAK-243 at 0.25, 0.5, or 10 μM for 3 h and subsequently infected with 200 MOI vp/cell of recombinant HBV expressing *Gaussia* luciferase. Markers of HBV infection include *Gaussia* luciferase (A,D), total HBV DNA (B,E), and cccDNA (C,F). Data are means ± SD, shown relative to 0 μM condition of TAK-243, analyzed by ANOVA followed by Dunnet's test: ns, not significant, **p* <0.05, ***p* <0.01, *****p* <0.0001. cccDNA, covalently closed circular DNA; MOI, multiplicity of infection; NTCP, sodium taurocholate cotransporting peptide; vp, viral particle.

Affiliations

[1]*Tytgat Institute for Liver and Intestinal Research, Amsterdam University Medical Centers, University of Amsterdam, Amsterdam, The Netherlands;* [2]*Amsterdam Gastroenterology, Endocrinology and Metabolism (AGEM), Amsterdam University Medical Centers, The Netherlands;* [3]*Institute of Virology, Technical University of Munich/Helmholtz Munich, Munich, Germany;* [4]*German Center for Infection Research (DZIF), Munich Partner Site, Munich, Germany*

Abbreviations

cccDNA, covalently closed circular DNA; DENV, Dengue virus; EGFR, epidermal growth factor receptor; FCS, fetal calf serum; HRP, horseradish peroxidase; K, lysine; MESNA, 2-mercaptoethanesulfonic acid sodium salt; MOI, multiplicity of infection; NTCP, sodium taurocholate cotransporting polypeptide; PEG, polyethylene glycol; PKC, protein kinase C; PNGase F, peptide N-glycosidase F; PrP, major prion protein; R, arginine; rHBV-Gaussia, recombinant HBV encoding Gaussia luciferase; S/Co, ratio of sample signal to internal cut-off signal; TBS-T, TBS-Tween; TCA, taurocholate; vp, viral particle; WT, wild-type.

Financial support

SFJvdG is supported by the Netherlands Organization for Scientific Research (VIDI 91713319 and Vici 09150182010007) and the European Research Council (Starting grant 337479). UP is supported by the German Research Foundation (DFG) TRR179, TP14 (project No. 272983813), and the German Center for Infection Research (DZIF, TTU05-820).

Conflicts of interest

The authors declare that there are no competing interests associated with the manuscript.

Please refer to the accompanying ICMJE disclosure forms for further details.

Authors' contributions

Concept and design of the experiments: MDA, TAN, UP, CCP, SFJvdG. Experiments and procedure: MDA, TAN, AO, AL. Writing – original draft: MDA, TAN, CCP, SFJvdG. Writing – review and editing: all authors.

Data availability

All data files are available upon request.

Acknowledgements

We would like to thank Claudia Sachetto and Giustiniano V.A. van Kerster for their technical assistance and Noam Zelcer for providing ubiquitin constructs.

Supplementary data

Supplementary data to this article can be found online at https://doi.org/10.1016/j.jhepr.2025.101534.

References

Author names in bold designate shared co-first authorship.

[1] Polaris Observatory Collaborators. Global prevalence, treatment, and prevention of hepatitis B virus infection in 2016: a modelling study. Lancet Gastroenterol 2018;3(6):383–403.
[2] Braga WSM, de Oliveira CMC, de Araújo JR, et al. Chronic HDV/HBV co-infection: predictors of disease stage - a case series of HDV-3 patients. J Hepatol 2014;61(6):1205–1211.
[3] Wedemeyer H, Schöneweis K, Bogomolov P, et al. Safety and efficacy of bulevirtide in combination with tenofovir disoproxil fumarate in patients with hepatitis B virus and hepatitis D virus coinfection (MYR202) : a multicentre, randomised, parallel-group, open-label, phase 2 trial. Lancet Infect Dis 2023;23(1):117–129.
[4] Yan H, Zhong GC, Xu GW, et al. Sodium taurocholate cotransporting polypeptide is a functional receptor for human hepatitis B and D virus. Elife 2012;1:e00049.
[5] Appelman MD, Wettengel JM, Protzer U. Molecular regulation of the hepatic bile acid uptake transporter and HBV entry receptor NTCP. BBA-Mol Cell Biol L 2021;1866(8):158960.
[6] Liu R, Chen C, Xia X, et al. Homozygous p.Ser267Phe in SLC10A1 is associated with a new type of hypercholanemia and implications for personalized medicine. Sci Rep 2017;7(1):9214.
[7] Binh MT, Hoan NX, Tong HV, et al. NTCP S267F variant associates with decreased susceptibility to HBV and HDV infection and decelerated progression of related liver diseases. Int J Infect Dis 2019;80:147–152.
[8] **Peng L**, **Zhao Q**, **Li Q**, et al. The p.Ser267Phe variant in SLC10A1 is associated with resistance to chronic hepatitis B. Hepatology 2015;61(4):1251–1260.
[9] Chuaypen N, Tuyapala N, Pinjaroen N, et al. Association of NTCP polymorphisms with clinical outcome of hepatitis B infection in Thai individuals. BMC Med Genet 2019;20.
[10] Shi J, Wang X, Qi W, et al. Association between NTCP hepatic expression and inflammation/fibrosis as well as gender-specific differences in chronic HBV-infected patients. J Med Virol 2024;96(1):e29428.
[11] Dye BT, Schulman BA. Structural mechanisms underlying posttranslational modification by ubiquitin-like proteins. Annu Rev Biophys Biomol Struct 2007;36:131–150.
[12] Rotin D, Staub O, Haguenauer-Tsapis R. Ubiquitination and endocytosis of plasma membrane proteins: role of Nedd4/Rsp5p family of ubiquitin-protein ligases. J Membr Biol 2000;176(1):1–17.
[13] Kuhlkamp T, Keitel V, Helmer A, et al. Degradation of the sodium taurocholate cotransporting polypeptide (NTCP) by the ubiquitin-proteasome system. Biol Chem 2005;386(10):1065–1074.
[14] Appelman MD, Chakraborty A, Protzer U, et al. N-glycosylation of the Na+-taurocholate cotransporting polypeptide (NTCP) determines its trafficking and stability and is required for hepatitis B virus infection. PLoS One 2017;12(1):e0170419.
[15] Lucifora J, Arzberger S, Durantel D, et al. Hepatitis B virus X protein is essential to initiate and maintain virus replication after infection. J Hepatol 2011;55(5):996–1003.
[16] Bockmann JH, Xia Y, Stadler D, et al. Type III interferons induce cccDNA degradation similar to type I interferons in HBV-infected HepaRG cells. Z Gastroenterol 2015;53:A5–A54.
[17] Bijsmans IT, Bouwmeester RA, Geyer J, et al. Homo- and hetero-dimeric architecture of the human liver Na^+-dependent taurocholate co-transporting protein. Biochem J 2012;441(3):1007–1015.
[18] Nelson JK, Cook EC, Loregger A, et al. Deubiquitylase inhibition reveals liver X receptor-independent transcriptional regulation of the E3 ubiquitin ligase IDOL and lipoprotein uptake. J Biol Chem 2016;291(9):4813–4825.
[19] Donkers JM, Zehnder B, van Westen GJP, et al. Reduced hepatitis B and D viral entry using clinically applied drugs as novel inhibitors of the bile acid transporter NTCP. Sci Rep 2017;7(1):15307.
[20] **Robin MJD**, **Appelman MD**, Vos HR, et al. Calnexin depletion by endoplasmic reticulum stress during cholestasis inhibits the Na^+-taurocholate cotransporting polypeptide. Hepatol Commun 2018;2(12):1550–1566.
[21] van de Graaf SFJ, Rescher U, Hoenderop JGJ, et al. TRPV5 is internalized via clathrin-dependent endocytosis to enter a Ca-controlled recycling pathway. J Biol Chem 2008;283(7):4077–4086.
[22] Koenderink JB, Geibel S, Grabsch E, et al. Electrophysiological analysis of the mutated Na,K-ATPase cation binding pocket. J Biol Chem 2003;278(51):51213–51222.
[23] Slijepcevic D, Roscam Abbing RLP, Katafuchi T, et al. Hepatic uptake of conjugated bile acids is mediated by both sodium taurocholate cotransporting polypeptide and organic anion transporting polypeptides and modulated by intestinal sensing of plasma bile acid levels in mice. Hepatology 2017;66(5):1631–1643.
[24] Wing PA, Davenne T, Wettengel J, et al. A dual role for SAMHD1 in regulating HBV cccDNA and RT-dependent particle genesis. Life Sci Alliance 2019;2(2):e201900355.
[25] Ko C, Chakraborty A, Chou WM, et al. Hepatitis B virus genome recycling and de novo secondary infection events maintain stable cccDNA levels. J Hepatol 2018;69(6):1231–1241.
[26] Park JH, Iwamoto M, Yun JH, et al. Structural insights into the HBV receptor and bile acid transporter NTCP. Nature 2022;606(7916):1027–1031.
[27] Guo WN, Zhu B, Ai L, et al. Animal models for the study of hepatitis B virus infection. Zool Res 2018;39(1):25–31.
[28] Lempp FA, Wiedtke E, Qu B, et al. Sodium taurocholate cotransporting polypeptide is the limiting host factor of hepatitis B virus infection in macaque and pig hepatocytes. Hepatology 2017;66(3):703–716.
[29] Muller SF, Konig A, Doring B, et al. Characterisation of the hepatitis B virus cross-species transmission pattern via Na+/taurocholate co-transporting polypeptides from 11 New World and Old World primate species. PLoS One 2018;13(6):e0199200.
[30] Urban S, Bartenschlager R, Kubitz R, et al. Strategies to inhibit entry of HBV and HDV into hepatocytes. Gastroenterology 2014;147(1):48–64.
[31] Ni Y, Lempp FA, Mehrle S, et al. Hepatitis B and D viruses exploit sodium taurocholate co-transporting polypeptide for species-specific entry into hepatocytes. Gastroenterology 2014;146(4):1070–1083.
[32] **Petersen J**, **Dandri M**, Mier W, et al. Prevention of hepatitis B virus infection in vivo by entry inhibitors derived from the large envelope protein. Nat Biotechnol 2008;26(3):335–341.
[33] Takeuchi JS, Fukano K, Iwamoto M, et al. A single adaptive mutation in sodium taurocholate cotransporting polypeptide induced by hepadnaviruses determines virus species specificity. J Virol 2019;93(5):e01432-18.
[34] Zakrzewicz D, Leidolf R, Kunz S, et al. Tyrosine 146 of the human Na^+/taurocholate cotransporting polypeptide (NTCP) is essential for its hepatitis B virus (HBV) receptor function and HBV entry into hepatocytes. Viruses 2022;14(6):1259.
[35] Yan H, Peng B, He W, et al. Molecular determinants of hepatitis B and D virus entry restriction in mouse sodium taurocholate cotransporting polypeptide. J Virol 2013;87(14):7977–7991.
[36] Miranda M, Dionne KR, Sorkina T, et al. Three ubiquitin conjugation sites in the amino terminus of the dopamine transporter mediate protein kinase C-dependent endocytosis of the transporter. Mol Biol Cell 2007;18(1):313–323.
[37] Miranda M, Wu CC, Sorkina T, et al. Enhanced ubiquitylation and accelerated degradation of the dopamine transporter mediated by protein kinase C. J Biol Chem 2005;280(42):35617–35624.
[38] Sorkina T, Hoover BR, Zahniser NR, et al. Constitutive and protein kinase C-induced internalization of the dopamine transporter is mediated by a clathrin-dependent mechanism. Traffic 2005;6(2):157–170.
[39] Sorkina T, Miranda M, Dionne KR, et al. RNA interference screen reveals an essential role of Nedd4-2 in dopamine transporter ubiquitination and endocytosis. J Neurosci 2006;26(31):8195–8205.
[40] González-González IM, García-Tardón N, Giménez C, et al. PKC-dependent endocytosis of the GLT1 glutamate transporter depends on ubiquitylation of lysines located in a C-terminal cluster. Glia 2008;56(9):963–974.
[41] de Juan-Sanz J, Zafra F, Lopez-Corcuera B, et al. Endocytosis of the neuronal glycine transporter GLYT2: role of membrane rafts and protein kinase C-dependent ubiquitination. Traffic 2011;12(12):1850–1867.
[42] Fernandez-Sanchez E, Martinez-Villarreal J, Gimenez C, et al. Constitutive and regulated endocytosis of the glycine transporter GLYT1b is controlled by ubiquitination. J Biol Chem 2009;284(29):19482–19492.
[43] Stross C, Helmer A, Weissenberger K, et al. Protein kinase C induces endocytosis of the sodium taurocholate cotransporting polypeptide. Am J Physiol Gastrointest Liver Physiol 2010;299(2):G320–G328.
[44] Stross C, Kluge S, Weissenberger K, et al. A dileucine motif is involved in plasma membrane expression and endocytosis of rat sodium taurocholate cotransporting polypeptide (NTCP). Am J Physiol Gastrointest Liver Physiol 2013;305(10):G722–G730.
[45] Herrscher C, Pastor F, Burlaud-Gaillard J, et al. Hepatitis B virus entry into HepG2-NTCP cells requires clathrin-mediated endocytosis. Cell Microbiol 2020;22(8):e13205.

[46] Huang HC, Chen CC, Chang WC, et al. Entry of hepatitis B Virus into immortalized human primary hepatocytes by clathrin-dependent endocytosis. J Virol 2012;86(17):9443–9453.
[47] Iwamoto M, Saso W, Nishioka K, et al. The machinery for endocytosis of epidermal growth factor receptor coordinates the transport of incoming hepatitis B virus to the endosomal network. J Biol Chem 2020;295(3):800–807.
[48] Dejarnac O, Hafirassou ML, Chazal M, et al. TIM-1 ubiquitination mediates Dengue virus entry. Cell Rep 2018;23(6):1779–1793.

Keywords: SLC10A1/NTCP; HBV; Liver; Myrcludex B; Endocytosis; Transport; Ubiquitin.
Received 9 October 2024; received in revised form 10 July 2025; accepted 21 July 2025; Available online 31 July 2025

Journal of Hepatology, Volume *7*

Supplemental information

NTCP ubiquitination enables HBV infection

Monique D. Appelman, Thuc-Anh Nguyen, Andreas Oswald, Aaron Lucko, Coen C. Paulusma, Ulrike Protzer, and Stan F.J. van de Graaf

NTCP ubiquitination enables HBV infection

Monique D. Appelman, Thuc-Anh Nguyen, Andreas Oswald, Aaron Lucko, Coen C. Paulusma, Ulrike Protzer, Stan F.J. van de Graaf

Table of contents

Fig. S1……………………………………………………………………………………….2

Fig. S2……………………………………………………………………………………….4

Fig. S3……………………………………………………………………………………….5

Fig. S4……………………………………………………………………………………….7

Fig. S5……………………………………………………………………………………….8

Fig. S6……………………………………………………………………………………….9

Fig. S7……………………………………………………………………………………...10

Table S1……………………………………………………………………………………11

Table S2……………………………………………………………………………………11

A

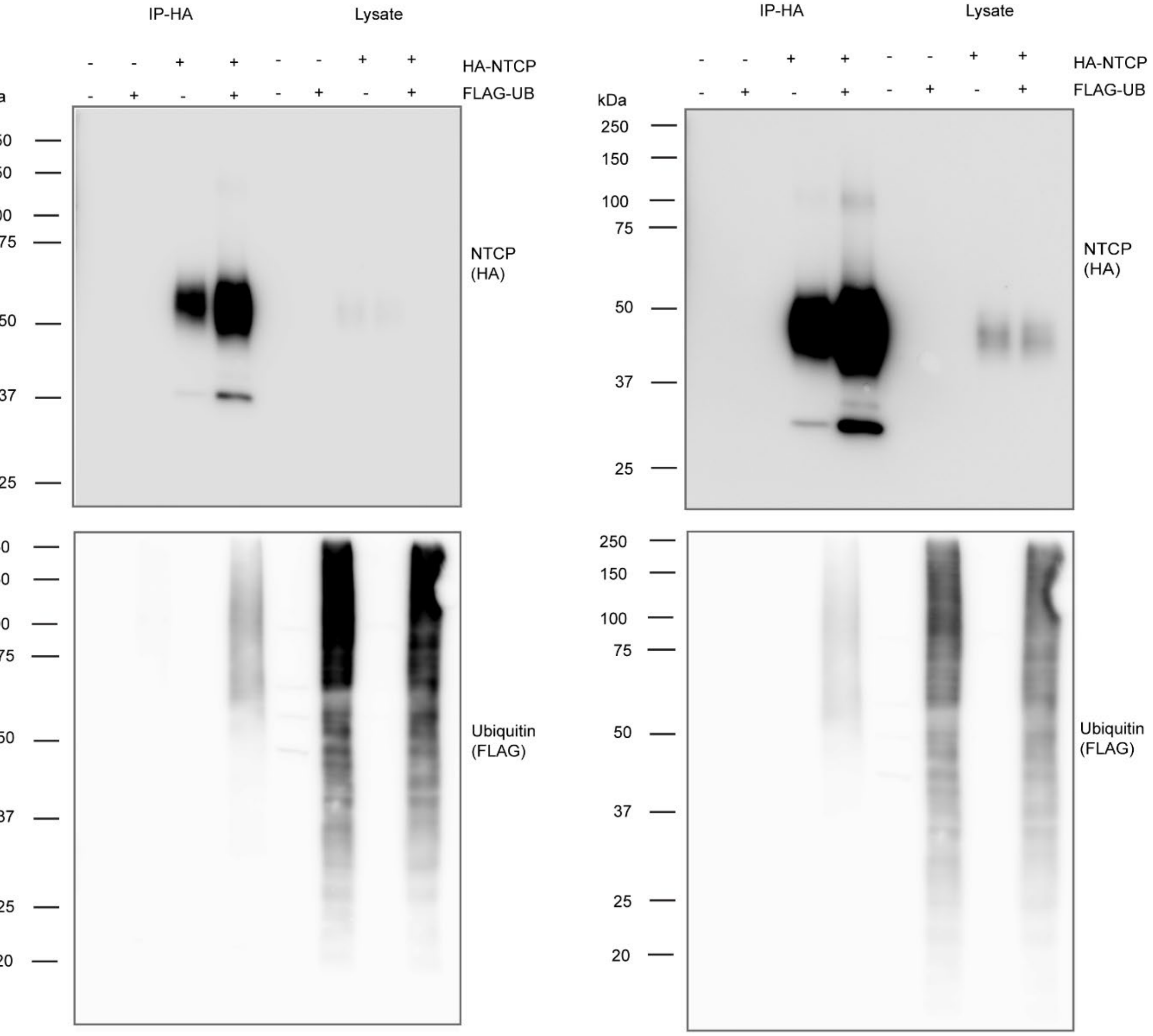

IP-HA
Lysate
HA-NTCP
FLAG-UB
kDa
250
150
100
75
50
37
25
20
NTCP (HA)
Ubiquitin (FLAG)

B

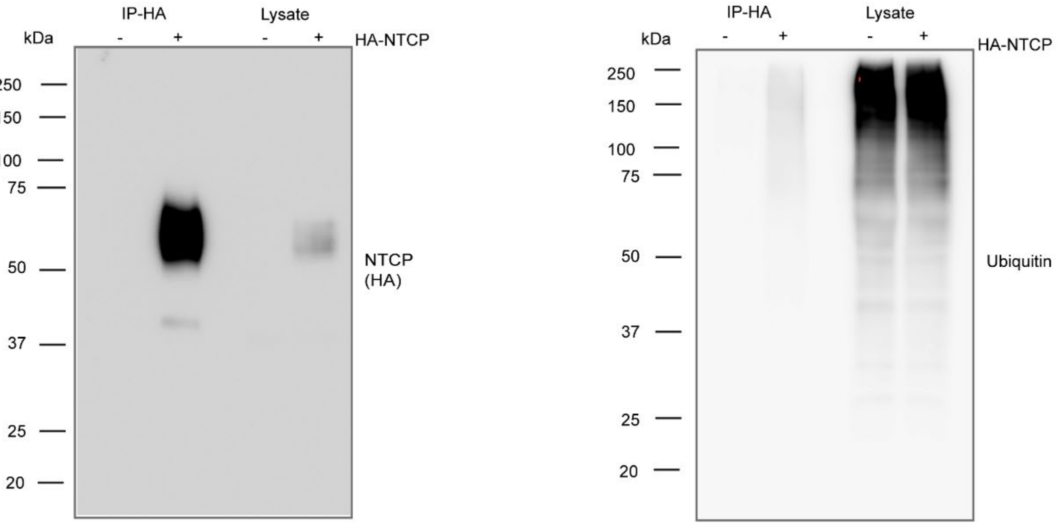

IP-HA
Lysate
HA-NTCP
kDa
250
150
100
75
50
37
25
20
NTCP (HA)
Ubiquitin

Fig. S1. Complete uncropped western blots of Fig. 1. (A) Complete western blot of Fig. 1A showing FLAG-Ubiquitin co-precipitation with HA-NTCP in U2OS cells stably expressing HA-tagged NTCP and transient overexpressing FLAG-tagged ubiquitin in two different light intensities. (B) Complete western blot of Fig. 1B showing endogenous ubiquitin co-precipitation with HA-NTCP in U2OS cells.

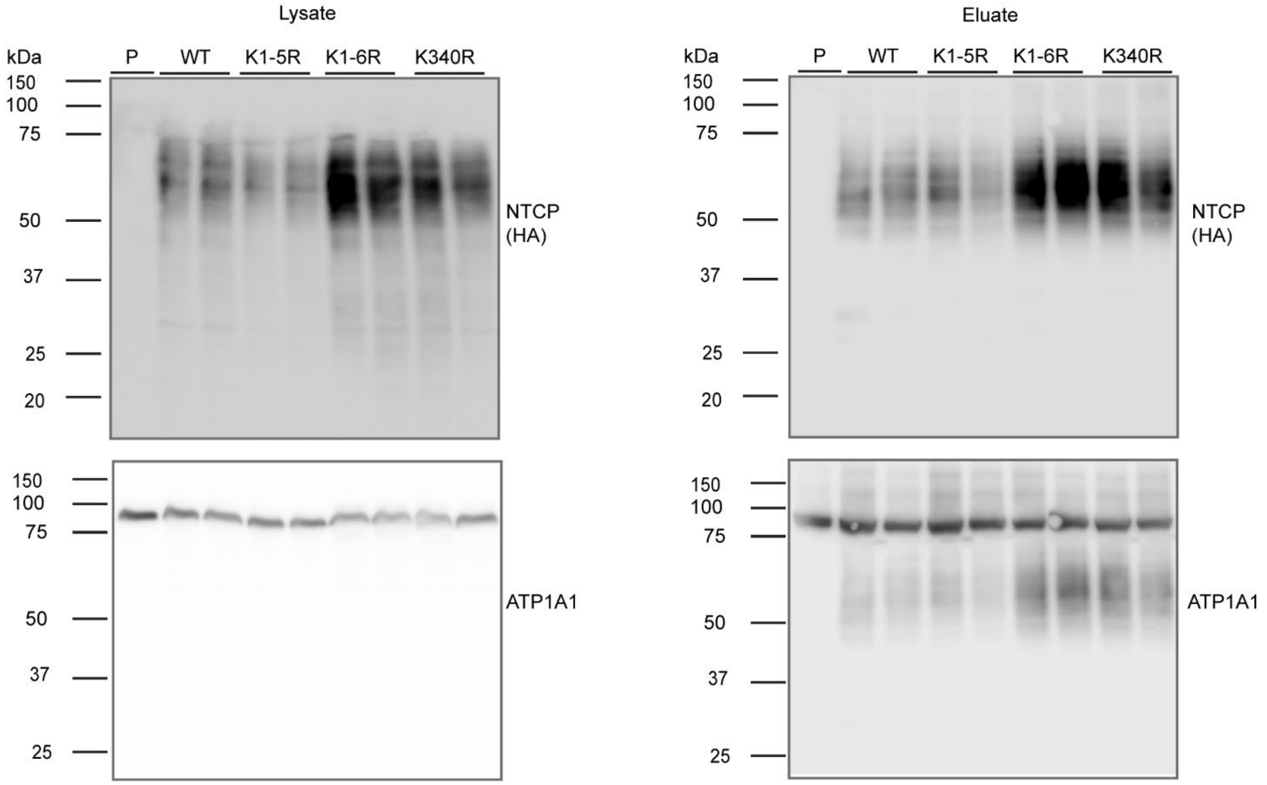

Fig. S2. Complete uncropped western blots of Fig.2. Complete western blot of Fig 2D showing immunoblot from a surface biotinylation experiment of HepG2 cells stably expressing $NTCP^{WT}$, $NTCP^{K1\text{-}5R}$, $NTCP^{K1\text{-}6R}$ and $NTCP^{K340R}$ showing NTCP protein levels in total lysate (left) and at the plasma membrane (right). ATP1A1 was included as a transfer control. ‘P’ denotes parental cells without HA-NTCP overexpression.

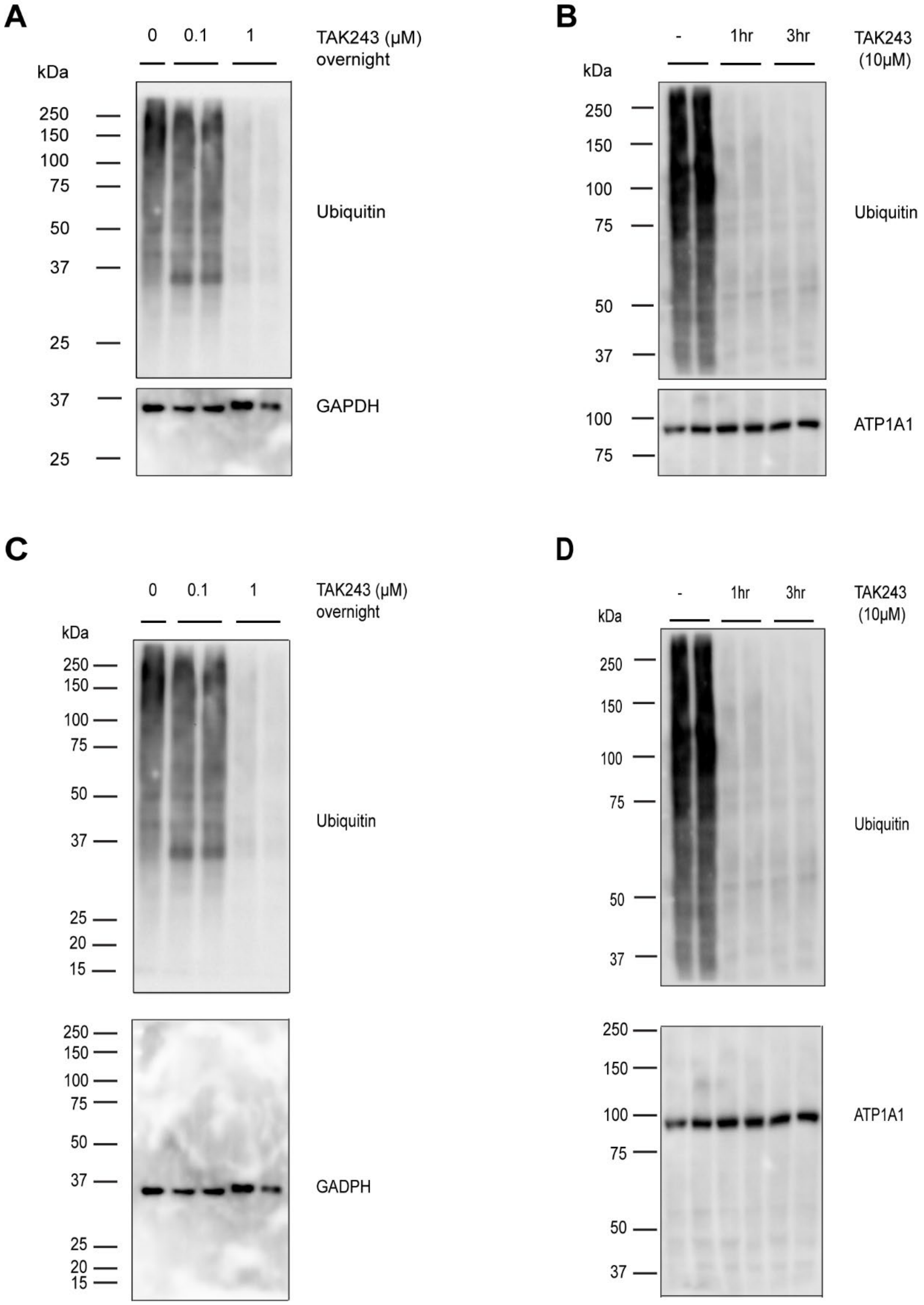

Fig. S3. Ubiquitination inhibition with TAK-243. Cropped (A, B) and uncropped (C, D) western blots of HepG2 cells overexpressing NTCP^{WT} treated withTAK-243. Overnight treatment of TAK-243 reduces ubiquitination at 0.1 µM but most efficiently at 1 µM (A, C).

At a high concentration of 10 µM, TAK-243 reduces ubiquitination markedly after 1-3 hours of treatment (B, D).

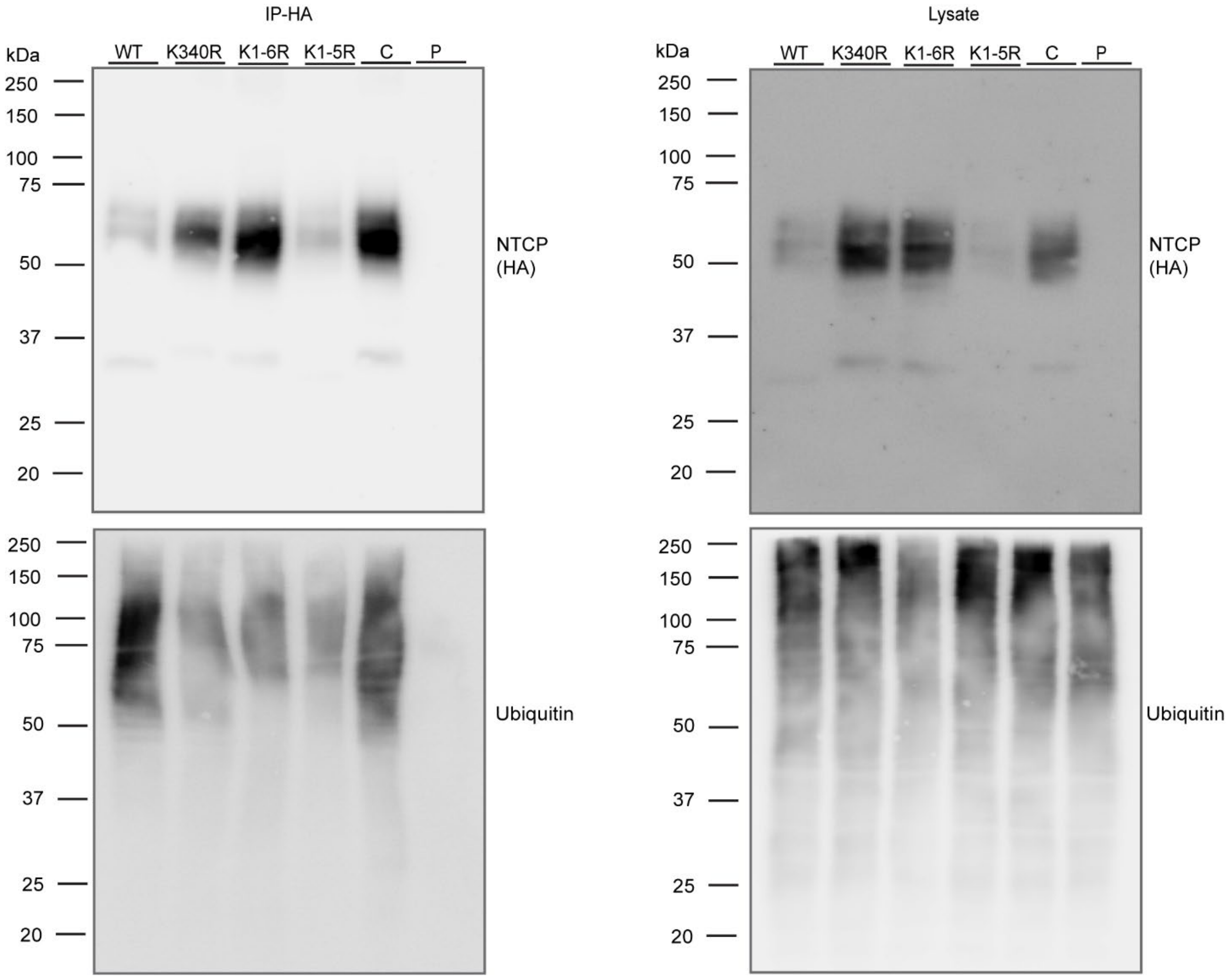

Fig. S4. Complete uncropped western blot of Fig 2G. Complete western blot of Fig. 2G showing immunoprecipitation of HA-NTCP in U2OS cells stably expressing HA-NTCP^{WT} or HA-NTCP^{K340R}, NTCP$^{K1\text{-}5R}$, NTCP$^{K1\text{-}6R}$, HA-NTCP (C) or parental U2OS (P), which shows that co-precipitation of ubiquitin in HA-NTCP^{K340R} cells was strongly reduced compared to HA NTCP^{WT} cells. The HA-NTCP (C) serves as a positive control in this experiment. Western blot is cropped and flipped horizontally to generate Fig. 2G.

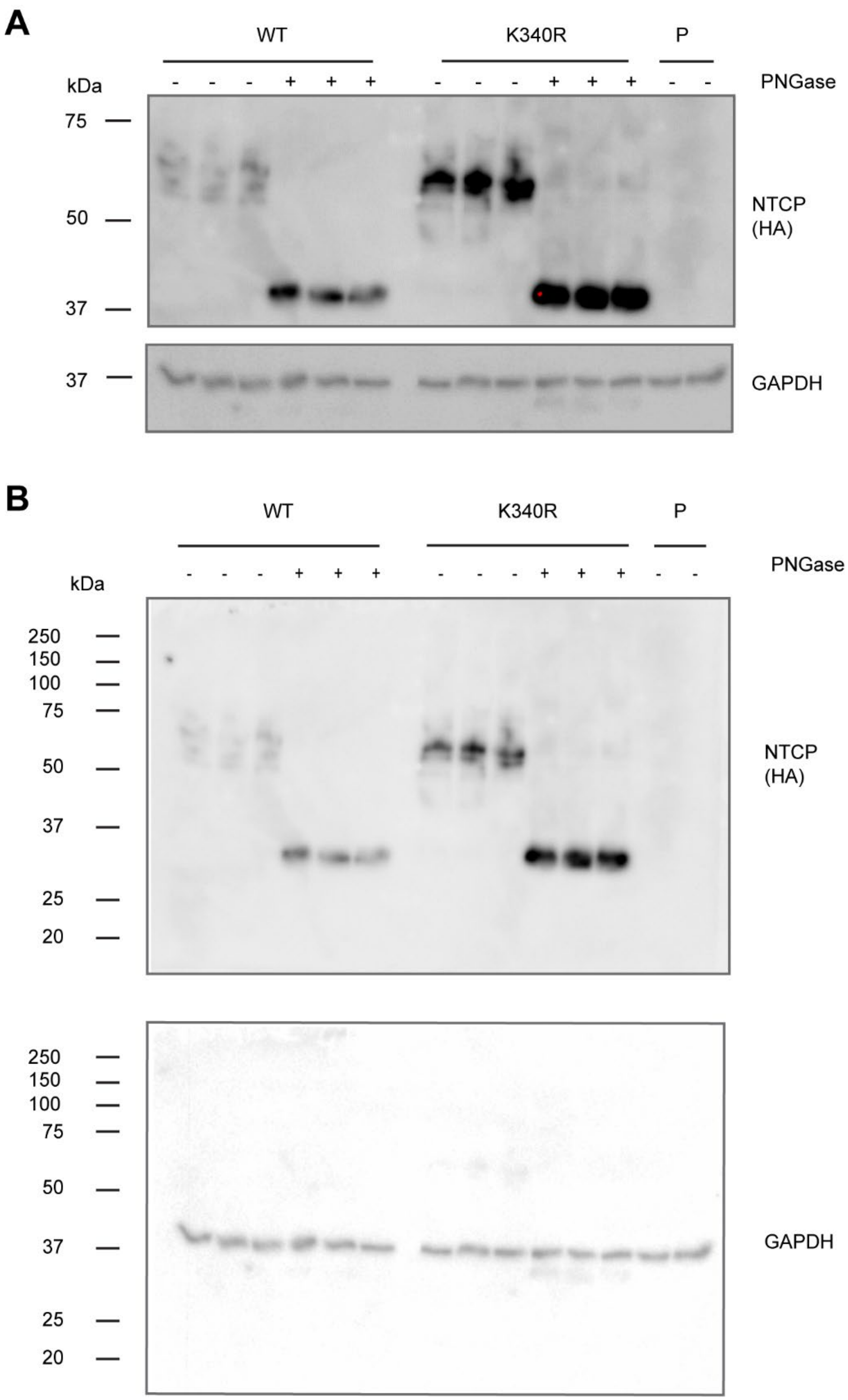

Fig. S5. The K340R mutation does not affect NTCP glycosylation. Cropped (A) and complete uncropped (B) western blots of lysates of HepG2 cells overexpressing $NTCP^{WT}$ or $NTCP^{K340R}$ or parental treated with PNGaseF for 2h at 37°C (500 units) prior to immunoblotting for NTCP.

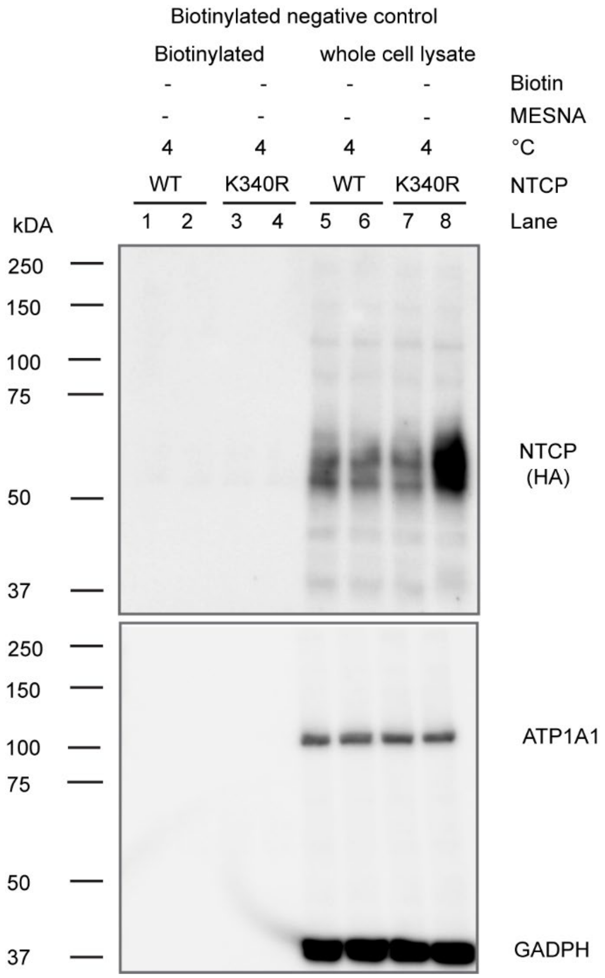

Fig. S6. Negative controls for biotinylation. Non-biotinylated fractions of HepG2 cells stably expressing $NTCP^{WT}$ or $NTCP^{K340R}$ were quantified**.** The omission of biotin prevented the precipitation of NTCP while in whole-cell lysate, NTCP was detected.

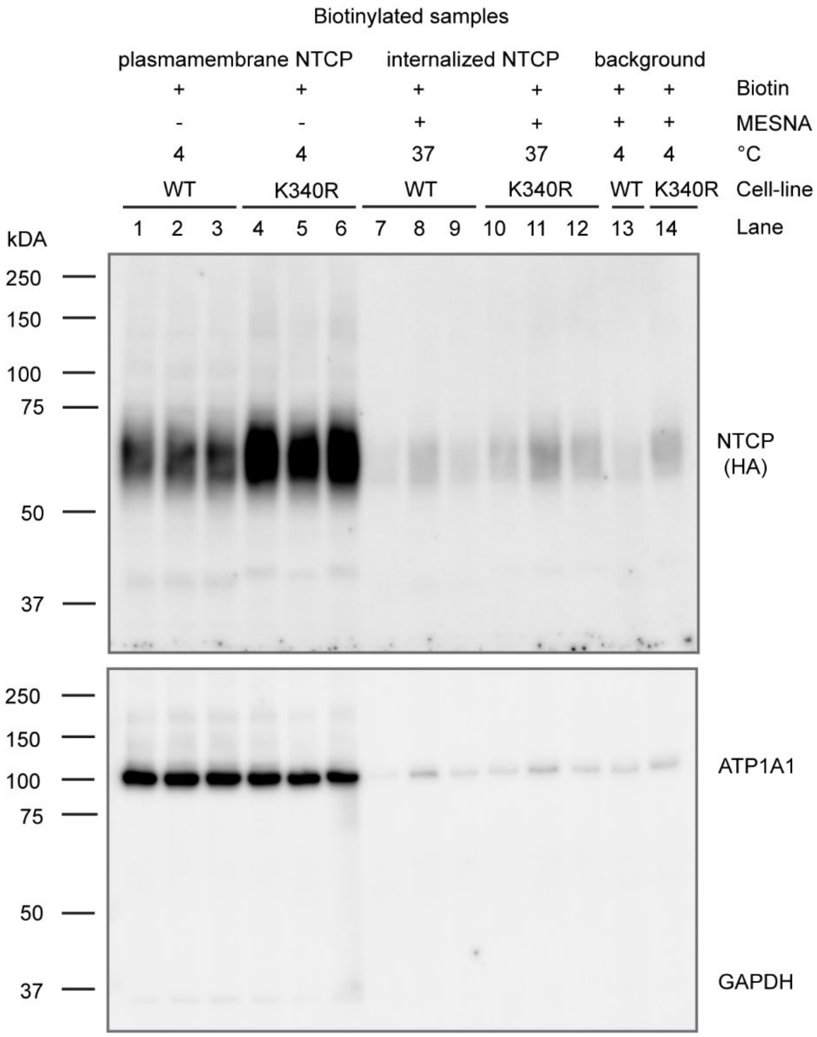

Fig. S7. Complete uncropped western blot of Fig. 3B. Endocytosis of $NTCP^{WT}$ and $NTCP^{K340R}$ in HepG2 cells was examined using biotin pulse-chase labeling of plasma membrane proteins. Total plasma membrane NTCP was determined from biotinylated cells kept at 4°C. To quantify internalized NTCP, cells were incubated at 37°C for 1.5 hour to allow endocytosis of biotinylated NTCP followed by MESNA treatment to remove biotin remaining on the plasma membrane. Additionally, biotinylated cells kept at 4°C were treated with MESNA to examine MESNA efficiency and quantify background fractions.

Supplementary Tables

Table S1. Primer pairs used for generating HA-hNTCP lysine mutants

Construct	Primers
HA-hNTCP^{K1-5R}	Fw: TGGTGCTATGAGAGATTCAGGACTCCCAGGGATAGAACAAGAATGATCTACACA<br>Rv: TGTGTATAGCATTCTTGTTCTATCCCTGGGAGTCCTGAATCTCTCATAGCACCA
HA-hNTCP^{K340R}	Fw: GCTCTGGGAAATGGCACCTACAGAGGGAGGACTGCTCC<br>Rv: GGAGCAGTCCTCCCCTCTGTAGGTGCCATTTCCCAG
Seq-hNTCP 1	CATGAAGGGGGACATGAACCTC
Seq-hNTCP 2	TGATGCCTTTTATTGGCTTT

Table S2. Primers used for qRT-PCR

Gene	Primers
NTCP	Fw: GGACATGAACCTCAGCATTGTG<br>Rv:GCCGTTTGGATTTGAGGACG
36B4	Fw:TCATCAACGGTACAAACGA<br>Rv: GAACGACTTTTCCAGTTCCG
HBV cccDNA	Fw: GACTCTCTCGTCCCCTTCTC<br>Rv: ATGGTGAGGTGAACAATGCT
HBV DNA, rcDNA	Fw: GTTGCCCGTTTGTCCTCTAATTC<br>Rv: GGAGGGATACATAGAGGTTCCTTGA
PrP	Fw: TGCTGGGAAGTGCCATGAG<br>Rv: CGGTGCATGTTTTCACGATAGTA
